# Self-awareness, self-regulation, and self-transcendence (S-ART): a framework for understanding the neurobiological mechanisms of mindfulness

**DOI:** 10.3389/fnhum.2012.00296

**Published:** 2012-10-25

**Authors:** David R. Vago, David A. Silbersweig

**Affiliations:** Functional Neuroimaging Laboratory, Department of Psychiatry, Brigham and Women's HospitalBoston, MA, USA

**Keywords:** mindfulness, meditation, contemplative, self-regulation, self-awareness, self, brain networks

## Abstract

Mindfulness—as a state, trait, process, type of meditation, and intervention has proven to be beneficial across a diverse group of psychological disorders as well as for general stress reduction. Yet, there remains a lack of clarity in the operationalization of this construct, and underlying mechanisms. Here, we provide an integrative theoretical framework and systems-based neurobiological model that explains the mechanisms by which mindfulness reduces biases related to self-processing and creates a sustainable healthy mind. Mindfulness is described through systematic mental training that develops meta-awareness (self-awareness), an ability to effectively modulate one's behavior (self-regulation), and a positive relationship between self and other that transcends self-focused needs and increases prosocial characteristics (self-transcendence). This framework of self-awareness, -regulation, and -transcendence (S-ART) illustrates a method for becoming aware of the conditions that cause (and remove) distortions or biases. The development of S-ART through meditation is proposed to modulate self-specifying and narrative self-networks through an integrative fronto-parietal control network. Relevant perceptual, cognitive, emotional, and behavioral neuropsychological processes are highlighted as supporting mechanisms for S-ART, including intention and motivation, attention regulation, emotion regulation, extinction and reconsolidation, prosociality, non-attachment, and decentering. The S-ART framework and neurobiological model is based on our growing understanding of the mechanisms for neurocognition, empirical literature, and through dismantling the specific meditation practices thought to cultivate mindfulness. The proposed framework will inform future research in the contemplative sciences and target specific areas for development in the treatment of psychological disorders.

“*To study the Way is to study the self. To study the self is to forget the self. To forget the self is to be enlightened by all things. To be enlightened by all things is to remove the barriers between one's self and others*.”

(Dogen, 2002)

## Introduction

In the last two decades, the concept of mindfulness as a state, trait, process, and intervention has been successfully adapted in contexts of clinical health and psychology, especially with relation to treating stress and targeting emotion dysregulation. Operationalizing mindfulness has been somewhat challenging given the plurality of cultural traditions from which the concept originates, the difficulty with which it is measured, and its distinction from its common usage [see Baer (2003); Dimidjian and Linehan (2003); Brown and Ryan (2004); Grossman (2008); Gethin (2011)].

Generally speaking, there are two models for cultivating mindfulness in the context of meditation practice—a 2500-year old historical model that is rooted in Buddhist science and a 25-year old contemporary model that is heavily influenced by Jon Kabat-Zinn's Mindfulness-Based Stress Reduction (MBSR) course, an adaptation of specific Buddhist techniques intended for general stress reduction (Kabat-Zinn, 1990). The historical model for training the mind has similar goals to the contemporary western medical model: both are interested in reducing suffering, enhancing positive emotions, and improving quality of life.

Although the contemporary view of the concept, “mindfulness” is increasingly becoming part of popular culture, there remains no single “correct” or “authoritative version” of mindfulness and the concept is often trivialized and conflated with many common interpretations. Mindfulness is described as (1) A temporary state of non-judgmental, non-reactive, present-centered attention and awareness that is cultivated during meditation practice; (2) An enduring trait that can be described as a dispositional pattern of cognition, emotion, or behavioral tendency; (3) A meditation practice; (4) An intervention. Dispositional mindfulness is now measured by at least eight self-report scales that are often uncorrelated with each other (Grossman and Van Dam, 2011). These semantic differences are problematic in the laboratory setting. Here we attempt to address this conceptual problem by synthesizing a comprehensive conceptual framework of self-processing in the context of neurobiological mechanisms by which mindfulness functions and that focuses on the goals of mindfulness-based meditation practice: to reduce suffering and create a sustainable healthy mind.

The proposed framework for understanding mindfulness focuses on self-processing and the underlying neural systems involved in self-awareness, -regulation, and -transcendence (S-ART). Different approaches to understanding mindfulness may focus on one aspect more than another—S-ART attempts to synthesize a unified framework that integrates the traditional Buddhist and contemporary models. The S-ART framework operates using the underlying premise that our perception, cognitions, and emotions related to our ordinary experiences can be distorted or biased to varying degrees. Depending on certain dispositional factors, these biases are sometimes pathological, but exist on a spectrum and may therefore be present without any clear psychopathology. Within this framework, mindfulness is described to reduce such biases through specific forms of mental training that develop meta-awareness of self (self-awareness), an ability to effectively manage or alter one's responses and impulses (self-regulation), and the development of a positive relationship between self and other that transcends self-focused needs and increases prosocial characteristics (self-transcendence). In support of S-ART, six neurocognitive component mechanisms that are highly integrated and strengthened together through intentional mental strategies underlying the practice and cultivation of mindfulness are proposed to modulate networks of self-processing and reduce bias. These mechanisms include intention and motivation, attention and emotion regulation, extinction and reconsolidation, prosociality, non-attachment, and de-centering. Thus, rather than reducing mindfulness down to a unitary dimension, the S-ART describes mindfulness in a broader framework of perceptual, physiological, cognitive, emotional, and behavioral component processes.

## Operationalizing mindfulness—integrating the historical and contemporary perspectives

### Defining meditation and mindfulness from the historical perspective

In the historical Buddhist context, the term *meditation* is used to translate the Sanskrit term *bhävana* and its Tibetan equivalent *sgoms*. Etymologically, the Sanskrit term connotes the notion of “cultivation,” or “causing to become” and the Tibetan equivalent, refers to “development of familiarity” (Thera, 1962; Rahula, 1974; Bodhi, 1999; Jinpa, 2009). In light of these definitions, it should be clear that a traditional emphasis of most meditation practice is that of mental development, in which the practitioner is cultivating a general sense of well-being and virtue along with a level of deep familiarity with one's inner mental landscape, and one's patterns of behavior (i.e., nature of mind) (Rahula, 1974; Bodhi, 1999; Wallace, 2011).

One of the original translations of sati into the English word, mindfulness, was by Davids (1882). It was translated from the Pali root, *sati* (Sanskrit: *smṛti*), literally meaning “memory,” and closely related to the verb, *sarati*, referring to the process, “to remember.” Most conceptualizations of mindfulness from the Buddhist perspective emphasize a close and constant connection between the functions of memory and attention (Thera, 1962). In fact, on closer examination, mindfulness can be described as the continuous discriminative attentional capacity for encoding and recollecting experiences efficiently—without forgetfulness or distraction, and in the appropriate context (Thera, 1962; Analayo, 2003; Wallace, 2006); however, from the classical Buddhist context, views on the concept of mindfulness vary considerably (Dreyfus, 2011; Dunne, 2011). The Satipatthāna Sutta, one of the most influential Buddhist texts, describes the practice of mindfulness as a direct path to the “cessation of suffering,” and as a fundamental quality or skill amongst a set of mental qualities developed through specific meditation practices (Analayo, 2003). So as to avoid confusion, we refer to the attentional skill here as, “mindful awareness.”

**Figure 1:**
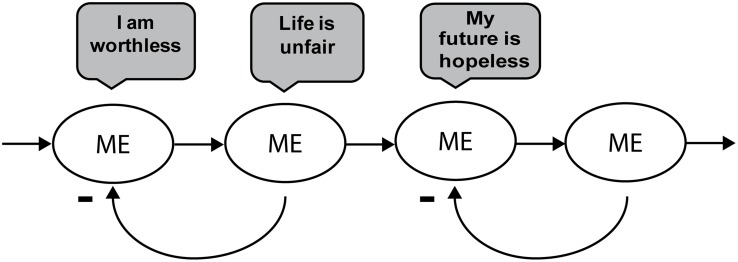
**Reification of the dysfunctional narrative self based on Beck's cognitive triad—a model for psychopathology (Beck, 1976).** Dysfunctional attitudes, rumination, and negative self-focus reify the self-narrative for the past, present, and future. The arrows depict causal influences for integrating self-identity (“ME”) over time and negative feedback in a dysfunctional narrative that leads to affect-biased attention at both sensory-perceptual and cognitive levels.

In the Buddhist context, suffering (Pali: *dukkha*) is related to a lack of awareness for the following fundamental characteristics of experience: (1) Habitual craving or attachment (to sensory/mental objects we like) and/or aversion (to sensory/mental objects we don't like); (2) All phenomena (including the concept of self) are impermanent (they arise and pass away). The characteristics are thought to be based on an inflated sense of self-importance or self-loathing (Thera, 1962). These characteristics of suffering are incorporated into the more contemporary model of suffering illustrated in Figure 1, in which habitual information processing biases reify a dysfunctional self-schema. In order to reduce suffering, the path of mindfulness is described to specifically place great emphasis on four particular tightly coupled qualities or skills which are developed through the prescribed meditative techniques, including (1) A balanced intensity of effort and diligence (Pali: *ātāpi*), (2) Wisdom of clear discernment or phenomenal clarity (Pali: *sampajaňa*), (3) Mindful awareness, and (4) Freedom from desire and discontent (Pali: *vineyya loke abhijjhā-domanassạm*), a form of equanimity. Equanimity (Pali, *upekkhā*) is translated as “on-looking” or “watching things as they arise” and is described to involve a balance of arousal without hyperexcitability or fatigue (Buddhaghosa, 1991). The application of equanimity involves impartiality without bias or discrimination arising from a sense of detachment from the attraction or aversion to ongoing experience (Gunaratana, 2002; Wallace, 2006; Bodhi, 1999). Phenomenal clarity refers to the intensity (or perceptual acuity) in which each moment is experienced. Qualities like equanimity and clarity develop over time along with mindful awareness, while one learns to neither suppress nor fixate on what is arising in sensory experience moment to moment. In concert with the other three qualities, mindful awareness is thought to be critical for improving access and insight toward subject-object relations, such that the most fundamental nature of objects (including the self) is perceived “as they truly are,” without distortions or biases inherent in cognition (Thera, 1962; Brown and Ryan, 2004; Wallace, 2006). This undistorted form of insight or experiencing is also referred to as “bare attention,” perception without interpretation (Thera, 1962; Analayo, 2003). The four qualities, including mindful awareness, critically facilitate the development of an advanced self-monitoring system that is the first essential step to S-ART—gaining awareness of suffering as it is described herein. Mindful awareness is also described to be specifically applied in a comprehensive way across four domains of experience: toward the body, toward feelings/sensations or affective tone, one's current mental state, and toward the matrix of interrelationships amongst all phenomena arising in one's consciousness (Buddhaghosa, 1991; Analayo, 2003; Wallace, 2011). It is important for the reader to be clear that this historical description of mindful awareness be seen as a critical skill, amongst others, developed in the meditation practices outlined in Buddhist teachings. It is this combination of four qualities along with the four applications of mindfulness that provide the historical framework for mindfulness, as the path toward reduced suffering and realization. Thus, our current framework on S-ART reflects the qualities that are emphasized here in the historical perspective and outline a skill set of processes that co-arise with mindful awareness and help create a sustainable healthy mind.

Emphasis from most traditional texts has also been on ethical conduct (Pali: *Sila*) and ethical dimensions of mindfulness so that actions along the path of reduced suffering continually remain “wholesome” (Thera, 1962; Buddhaghosa, 1991; Bodhi, 1999). Ethical conduct is based on the conception of universal love and compassion for all living beings. This quality is reflected in self-transcendence and the quality with which one brings awareness to oneself and those around us. Furthermore, the ethical emphasis suggests the practitioner call to mind the various beneficial and detrimental mental states in existence so that one does not forget how particular patterns of behavior make one feel. Thus, one has further motivation to show sympathy and compassion for those around us who are experiencing negative mental states (Gethin, 2011). The ethical dimension is part of a constellation of positive qualities that is evidently also necessary for the advancement of the practitioner (Thera, 1962; Buddhaghosa, 1991).

The multi-dimensional skill set underlying the construct of mindfulness we are attempting to operationalize can be also be conceptually distinguished from the more familiar notion for the flip side of mindlessness as described in other contexts of contemporary usage. For example, The Oxford English Dictionary (3rd edition) defines the common usage of mindful (mindfulness) as, “full of care”; heedful, thoughtful, and references the opposite of “extreme carelessness” (Dictionary, 2002). Langer (1989) defines mindfulness as “sense of situational awareness,” and emphasizes that mindfulness refers to the active construction of new categories and meanings when one pays attention to the stimulus properties of primarily external situations. She also emphasizes that mindfulness can be attributed to cognitive flexibility, an executive function allowing for ease in switching set. While such definitions are distinctly different from the concept of mindfulness as we have described it above, there is likely overlapping psychological processes between them. There is also a common practice of “cherry picking” aspects of the meditation practices described and adding it to a clinical intervention or an education curriculum and calling it, “mindfulness.” Although there may be benefits to doing such modifications, there are conceptual dangers for scientific investigation of such modifications without qualifying the use of the term in the particular context which it is used.

### Meditation practices that cultivate mindfulness

Although there is a heterogeneity amongst styles of meditation practice, the S-ART framework focuses on the two core practices typically described as focused attention (FA), a type of concentrative practice and insight or open monitoring (OM), a type of receptive practice (Buddhaghosa, 1991) (for detailed description of the practices, see (Lutz et al., 2007, 2008b). The two practices are outlined such that once the practitioner is able to stabilize the mind, decrease mental proliferation (i.e., rumination), and develop a fully absorbed state of concentration on an object like the breath using FA, he/she can move on to OM and other more advanced receptive practices that have no object of focus, but rather are receptive to all other physical and mental phenomena that arise (Thera, 1962; Wallace, 2006). The cultivation of ethical qualities (e.g., loving-kindness, compassion, forgiveness) through specific forms of meditation practices are also described to supplement these two core practices (Goldstein, 1976; Wallace, 2006; Lutz et al., 2007; Salzberg, 2011), support explicit ethical development, and has accompanied most mindfulness-based interventions (MBIs). In combination, these meditation practices are thought to facilitate the development of S-ART. Each process model illustrated in section “Mindfulness-Based Mental Training: Methods for Adaptive Self-Functioning and Integrating Self-Specific Networks Supporting S-ART” describes the overlapping component processes that are mapped onto self-specific brain networks outlined for the S-ART framework.

It should be noted that some traditions have equated both insight and FA practice with mindfulness and have referred to these individual types of meditation styles as “mindfulness meditation” (Kabat-Zinn, 1990; Brown and Ryan, 2004; Cahn and Polich, 2006). There is no rule concerning the ability to achieve the desired resultant states through any particular order of learning these practices, or whether they are practiced simultaneously, or through the practice of one method alone. Wallace (2006) emphasizes that mindfulness is cultivated in Samatha practice, and applied in the practice of Vipassana (insight), although others (Lutz et al., 2007) would argue that mindfulness can be cultivated in both FA and OM practice, a position we take here. Samatha practice has 10 sequential stages of development (directed attention, continuous attention, resurgent attention, close attention, tamed attention, pacified attention, fully pacified attention, single-pointed attention, attentional balance, and finally samatha) that could foreseeably be examined independently as one progresses in the practice longitudinally. The stages are described at length by the eighth-century Indian Buddhist contemplative Kamalashila (Lama Xiv et al., 2001) and also by the Buddhist scholar Alan Wallace (Wallace, 2006). Only the first four stages would be considered a concentration practice, the later stages of practice correspond to deeper, more subtle states of profound stillness and absorption (Pali: *jhanas*). Such absorptive states cultivate an experience of non-duality between subject-object relations, is thought to be experienced with joy and elation, and is associated with strong decreases in craving (aversion) for positive (negative) states. Jhanas also have states of absorption that are progressive and potentially measurable through phenomenological changes in experience of the meditator. To achieve the 10th stage of samatha apparently requires an exceptionally high level of mastery, which could take years of training and upwards of 10,000 h of formal practice (Wallace, 2006). Other meditative practices can also be considered a concentrative practice. For example, transcendental meditation (TM) centers attention on the repetition of a mantra; however, the method places primary emphasis on the resultant states of continued practice related to an absence of concentrative effort and the development of a witnessing, thought-free “transcendental awareness” (Mahesh Yogi, 1995).

### Contemporary definitions of mindfulness

Contemporary psychology and psychiatry have adopted secularized forms of mindfulness practice as an approach for increasing awareness and responding skillfully to mental processes that contribute to emotional distress and maladaptive behavior (Kabat-Zinn, 1990; Bishop et al., 2004; Carmody, 2009). In mainstream clinical literature, mindfulness has been described as a form of attention that is purposeful, non-reactive, non-judgmental, and in the present moment (Kabat-Zinn, 1990; Baer, 2003; Bishop et al., 2004; Carmody, 2009). This conceptualization for mindfulness originally proposed by Kabat-Zinn has been successfully incorporated into a number of evidenced-based clinical interventions, namely MBSR (Kabat-Zinn, 1990), Mindfulness-based Cognitive Therapy (MBCT) for prevention of depression relapse (Segal et al., 2002), Mindfulness-based Relapse Prevention (MBRP) for prevention of substance use relapse in addiction (Bowen et al., 2010), Relaxation Response for cardiovascular health and wellness (Benson, 2000), Acceptance and Commitment Therapy (ACT) for a wide range of psychological problems (Hayes et al., 2006), and Dialectical Behavior Therapy (DBT) for the treatment of borderline personality disorder (Linehan, 1993). The successes of MBIs have been measured in terms of decreasing clinical symptoms and improving overall mental health and well-being. The changes are based on the general framework of a manualized 8-week program of meditation and gentle Hatha yoga training, including 26 h of formal instruction (8 classes/2.5-h/ea.), variable amount of meditation time at home (45 min/day; averages reported of 246 min/week), plus an all-day 6-h class during the 6 week (in some cases half-day) (Carmody and Baer, 2009). This contemporary form of mindfulness training is also encouraged to be used during ordinary activities like walking, standing, and eating (Kabat-Zinn, 1982; Baer, 2003). Although there has yet to be a systematic study of the relationship between the magnitude of measured outcomes and class time, a recent review of class contact hours and effect sizes found that reductions in number of in-class hours does not necessarily lead to compromised outcomes (Carmody and Baer, 2008). In fact, there have been reported improvements in clinical symptoms and executive function in short-term training of specific mindfulness-based practices as short as three 20 min sessions (Zeidan et al., 2010) and changes in white matter connectivity after 11 h of training (Tang et al., 2010).

In attempts to measure the psychotherapeutic outcomes of MBIs, metrics for quantifying states and traits related to the construct of mindfulness have been created. Dispositional mindfulness is currently measured by at least eight scales, items of which were originally generated by psychology faculty and graduate students reportedly familiar with the construct of mindfulness and mindfulness-based psychotherapies (Feldman et al., 2007; Cardaciotto et al., 2008). The scales include the Mindful Attention Awareness Scale Brown (MAAS) (Brown and Ryan, 2003), Southampton Mindfulness Questionnaire (SMQ) (Chadwick et al., 2008), Philadelphia Mindfulness Scale (PHLMS) (Cardaciotto et al., 2008), Toronto Mindfulness Scale (TMS) (Lau et al., 2006), Freiburg Mindfulness Inventory (FMI) (Walach et al., 2006), Revised Cognitive and Affective Mindfulness Scale (CAMS-R) (Feldman et al., 2007), Kentucky Inventory of Mindfulness Skills (KIMS) (Baer et al., 2004), and Five Facet Mindfulness Questionnaire (FFMQ) (Baer et al., 2006). The FFMQ has the advantage over other measures given that it was developed based on the items of five existing self-report measures. Factor analyses of these measures resulted in five facets of mindfulness including (1) An enhanced capacity for noticing or attending to internal and external experiences (*OBSERVING*); (2) An enhanced capacity for noting and labeling internal experiences (feelings, images, and thoughts; *DESCRIBING*); (3) An enhanced capacity for acting with present-centered awareness rather than on “automatic pilot”—lost in the past or the future (*acting with AWARENESS*); (4) An enhanced ability to take a non-evaluative, non-judgmental stance toward inner thoughts, images, and feelings and outer experiences (*NON-JUDGEMENT*); and (5) An enhanced ability to allow thoughts, images, and feelings to come and go without reacting to them or getting carried away by them (*NON-REACTIVITY*). These five facets have shown very consistent changes with meditation training and symptom improvement (Baer, 2011). Each of the measures has their respective internal validity; however serious methodological and conceptual issues remain in the interpretation of changes on the FFMQ and amongst all the self-report measures of mindfulness (see Grossman and Van Dam, 2011). For example, there is a strong possibility for semantic differences in understanding by responders, there is a variability in definitions of mindfulness from the Buddhist traditions and amongst the measures themselves, features of prosocial behavior or affective style transformation are not accounted for, and there is strong potential bias in inventory developers and responders (Grossman, 2008). In addition, many of these measures rely heavily on reverse-scored items in which an endorsement of the low end of a trait scale does not imply the strong presence of its opposite (Grossman and Van Dam, 2011). Although there is clearly benefit in using these self-report methods, the aforementioned critical issues should prevent clinical research from confirming the efficacy of traditional systems based on these self-report measures and provide caution in making claims about potential mechanisms (Carmody, 2009; Grossman and Van Dam, 2011). Lastly, in consideration of elucidating contemporary definitions of mindfulness, one should consider Jon Kabat–Zinn's original intention for using the term, “…(we used) the word *mindfulness* intentionally as an umbrella term to describe our work … By ‘umbrella term’ I mean that it is used in certain contexts as a place-holder for the entire dharma, that it is meant to carry multiple meanings and traditions simultaneously” (Kabat-Zinn, 2011).

To date, there have been relatively few attempts at operationalizing mindfulness into distinct cognitive-neuro-psycho-social processes or proposing a conceptual mechanistic model (see Baer, 2003; Brown and Ryan, 2003; Bishop et al., 2004; Shapiro et al., 2006; Baer et al., 2009; Carmody, 2009; Fletcher et al., 2010; Williams, 2010). These models have been mostly clinically oriented and all have emphasized four major themes common to many existing psychotherapeutic approaches [see Castonguay and Beutler (2005)]: (1) a present-centered orientation of awareness; (2) An attitude that consists of a constellation of positive state-like qualities (open-hearted, non-judgmental; accepting) toward thoughts and feelings; (3) a positive intention or motivational component for clinical change or spiritual incentive; (4) development of a form of decentering or psychological distancing from one's thoughts and emotions. More recently, Holzel et al. (2011a) identified very specific component mechanisms by which mindfulness functions, including attention and emotion regulation, body awareness, and a change in the perspective on the self. Herein, we expand upon these component processes by focusing on neural systems of self-processing and mapping neurocognitive processes that support mindfulness-based meditation practices onto such systems.

## Biased self-processing: a contemporary model for suffering

The common thread that ties the historical and contemporary models of mindfulness together in the S-ART framework is an element of suffering and a distorted or biased sense of self, one's relation to others, events, and the external world. In studies of attention to emotion in the general population or in a clinical population, bias has referred to the tendency or extent to which emotional stimuli with either a negative or positive valence may be processed differently in comparison to neutral material. Interestingly, one of the goals of mindfulness-based practice is to make no such distinction between positive, negative, or neutral valence and treat all incoming stimuli with impartiality and equipoise. In relation to self-processing, affect-biased attention is associated with distortions in initial attention allocation toward momentary experience and/or subsequent information processing that either follows immediately after an emotional stimulus or is associated with real or imagined stimuli from the past or distant future. The capture of perceptual resources by a given stimulus is influenced not only by the characteristics of the stimulus itself, but also by higher control systems acting upon the representations of stimuli. Attentional selection is therefore determined by the outcome of competition between these multiple and potentially “biased” representations of the world and of one's perception of self in relation to the world.

Through either dispositional or experiential factors, attentional bias can reflect facilitated attentional engagement toward (or away from) stimuli that are contextually self-relevant (e.g., threat for fear-related disorders), or a difficulty in disengagement from such stimuli (Cisler et al., 2009). While engagement refers to an enhanced tendency to orient and select a given stimulus or location for processing, disengagement refers to the process by which selection and facilitated processing is withdrawn or inhibited (Yiend, 2010). Facilitated engagement may translate to hypervigilance or enhanced monitoring and artificially increased perception of threat in everyday experiences, the consequence of which is that all threatening stimuli (real or imagined) attract attention, consuming resources and affecting sensory-perceptual systems before there is full subjective awareness (Mathews and Mackintosh, 1998). On the other hand, avoidance refers to both automatic and strategic forms of emotion regulation in which elaborative or evaluative processing is reduced and threat value is deflated. Disengagement delays are similar to what meditation practitioners refer to as “mental stickiness” and describes our natural tendency to dedicate resources to an object of engagement to the extent that few resources remain to capture any other pertinent environmental information until one is able to disengage (“let go”) and re-orient. Over time, hypervigilance, avoidance, and disengagement delays can become habitual, contextually self-relevant, and highly crystalized sensory-affective-motor scripts and schemas that dictate tendencies toward behavior. This form of affect-biased attention has shown to play a major role in causally influencing and maintaining disordered affective states such as anxiety and depression. For example, in one of the most accepted models of psychopathology, Beck (1964) originally proposed, “… the processing of external events or internal stimuli is biased and therefore systematically distorts the individual's construction of his or her experiences, leading to a variety of errors (e.g., overgeneralization, selective abstraction, personalization). Underlying these distorted interpretations are dysfunctional beliefs incorporated into relatively enduring cognitive structures or schemas” (Beck, 2008). Studies have demonstrated that individuals with either a diagnosed clinical disorder or a known vulnerability demonstrate affect-biased attention that is contextually self-relevant (Yiend, 2010). Individuals with such biases may not necessarily have apparent psychopathology, but more likely fall somewhere on a spectrum of function on which one extreme is pathological. Success of both cognitive and mindfulness-based therapy relies on the removal of such dysfunctional beliefs and distortions (through different means). The meditation practitioner, whom may not even be a risk for developing psychopathology likely has attentional or emotional biases that are more subtle to detect.

The model of a psychopathological self-schema is depicted in Figure 1 in which habitual negative beliefs about the self (including their personal world and future), become reified through the continuous reinforcement of a feedback loop of affect-biased attention influencing subjective and behavioral symptoms and vice-versa. Formation of dysfunctional attitudes incorporated within cognitive schemas produce automatized, efficient implicit memories, and motor programs that represent skewed information processing from early perceptual stages to the cognitive interpretation (Beck, 1976, 2008). When these maladaptive scripts and schemas are active repeatedly throughout one's daily life, it can lead to rumination or mental proliferation, in which a stream of mental events feed off each other with no connection to the original sense impression that initiated the stream of thought. Ruminative behavior is often characterized as reducing information processing capacity, producing general interference effects with ongoing task demands, and impacting ability to deploy top-down control (Yiend, 2010). This impact on information processing capacity also colors the emotional tone of one's ongoing experience, reducing the opportunity for awareness of one's thoughts or patterns of behavior in response to particular contextual triggers.

The reified representation of self depicted in Figure 1 has a distinct pattern of perceptual, physiological, cognitive, emotional, and somatic activity related to each context and each time in which the self is actively engaging with the external or internal world. At each repeated exposure to the individual contextual features of any biased self-schema, there is a hypothesized non-conscious pattern completion of the entire dysfunctional system that facilitates habitual forms of processing and blocks novel interpretations about oneself (e.g., positively framed memories and self-schemas) and the external sensory world (e.g., efficient engagement and disengagement). Pattern completion refers to the ability of a network to retrieve an entire previously stored output pattern from a partially presented input pattern, increasing synaptic efficacy (Marr, 1971). Thus, a vulnerability or risk for triggering psychopathology becomes progressively stronger over one's lifespan (i.e., repeated negative self-schema), suggesting a “kindling” effect, stoking a fire that makes the reified self more difficult to change. The reification of the dysfunctional narrative self (NS) illustrates the contemporary understanding of suffering and the S-ART framework provides mechanisms through which mindfulness practice can unravel the cycle of dysfunctional attitudes toward the self and toward one's relationship with the world.

## Networks for self-processing that support S-ART

The framework for the development of S-ART rests upon existing brain networks that support systems of self-processing. These systems are proposed to be subject to modulation through specific mechanisms cultivated by mindfulness-based meditation practices and which are identified in the following section “Mindfulness-Based Mental Training: Methods for Adaptive Self-Functioning and Integrating Self-Specific Networks Supporting S-ART.” The networks identified in the S-ART framework elaborates upon past conceptualizations of self (James, 1892; Damasio, 1999; Gallagher, 2000; Legrand, 2007; Northoff and Panksepp, 2008) and distinguishes between two functional aspects of self-specifying processes, (1) non-conscious sensory-affective-motor processing, referred to here as the experiential enactive self (EES); (2) an agentic, self-as-subject acting as awareness in the present moment, referred to as an experiential phenomenological self (EPS). The S-ART also distinguishes between self-specifying processes and self-related processes, which refer to the evaluative self-as-object, reflecting the autobiographical narrative reconstructed from the past or projected into the future. This self-reflective form of self-processing is referred to here as NS (Christoff et al., 2011). Enaction, refers to the sensorimotor coupling between the organism and the environment which guides embodied action (Varela et al., 1991). The EES is conceptualized similarly to the “the physical self” described by James (James, 1890) and the “the proto-self” described by Damasio; whereas, the EPS is described similarly as the “core-self” (Damasio, 1999). This framework is by no means an exhaustive account of the nature of self, but is rather a simplified parcellation that is relevant to scaffold our conceptual account of self-specific processing and is susceptible to influence by the practice of mindfulness.

**Figure 2:**
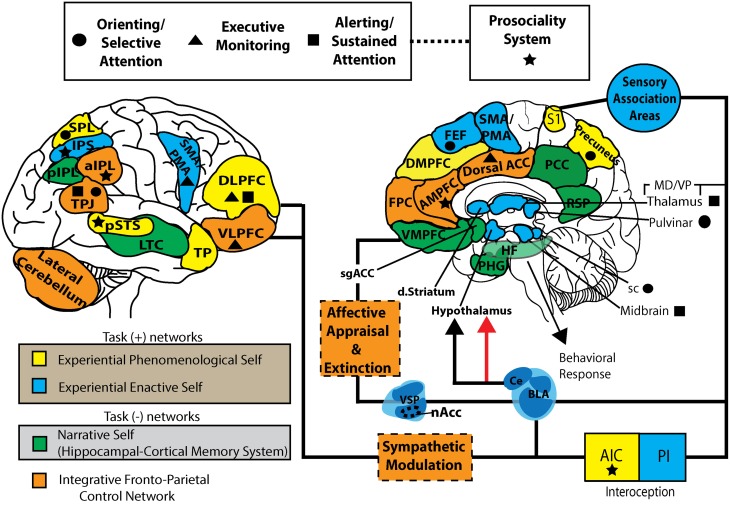
**Self-networks and neurocognitive systems supporting S-ART.** This working model represents a parcellation of task positive (self-specifying: EPS and EES), task negative (NS), and integrative fronto-parietal control networks. It also represents the individual components of the attentional systems and prosociality network purported to be modulated by mindfulness. The substrates for six component mechanisms of mindfulness within a framework of S-ART are also represented. EPS, experiential phenomenological self; EES, experiential enactive self; NS, narrative self; FPCN, fronto-parietal control network; FEF, frontal eye fields; DMPFC, dorsal-medial prefrontal cortex; AMPFC, anterior medial prefrontal cortex; VMPFC, ventromedial prefrontal cortex; PHG, parahippocampal gyrus; HF, hippocampal formation; RSP, retrosplenial cortex; PCC, posterior cingulate cortex; Dorsal ACC, dorsal anterior cingulate cortex; DLPFC, dorsolateral prefrontal cortex; VLPFC, ventrolateral prefrontal cortex; TP, temporal pole, LTC, lateral temporal cortex; TPJ, temporoparietal junction; sPL, superior parietal lobe; pIPL, posterior inferior parietal lobe; aIPL, anterior inferior parietal lobe; nAcc, nucleus accumbens; VSP, ventrostriatal pallidum; dstriatum, dorsal striatum; S1, primary somatosensory cortex; AIC, anterior insular cortex; PIC, posterior insular cortex; sgACC, subgenual anterior cingulate cortex; VMpo, ventromedial posterior nucleus; sc, superior colliculus; BLA, basolateral amygdala; CE, central nucleus.

There is now evidence for the existence of large scale neural networks for which the three mutually dependent systems of self referred to here can be mapped onto both functionally and with strong anatomical specificity. One caveat to consider in the interpretation of these networks is that many of the identified substrates do not have functional roles that are reducible to definitive functional categories, but are rather contextually dependent and operate on a dynamic, functional gradient which allows for some functional overlap. Networks underlying self-specifying (EES and EPS) and NS processes have specifically been shown to involve functionally distinct, and potentially competing, brain networks that can be broadly distinguished by their contrasting roles in primary modes of sensation-perception, and attention to the external world versus internally directed mentation involving long-term memory [see Buckner and Carroll (2007); Vincent et al. (2008)]. At the core of these two networks are the dorsal attention system (DAS) and the hippocampal-cortical memory system (HCMS), a component of the brain's default network (Vincent et al., 2008) (See Figure 2). The HCMS is described as a task negative network given its anticorrelation with goal-directed task performance; whereas, the networks identified as the EES and EPS have been identified as task-positive networks given its positive correlation with goal-directed activity (Fox et al., 2005; Broyd et al., 2009). Furthermore, several recent studies show that spontaneous activity within the DAS and the HCMS is negatively correlated—leading to their being described as anticorrelated brain systems (Fox et al., 2005; Vincent et al., 2008). The task-positive network is functionally parcellated here into the EPS and EES in order to account for the differences in phenomenological representation of experience that is primary sensory-perceptual awareness (i.e., EPS), and non-conscious processing (i.e., EES).

### The enactive experiential self (EES)

The EES network integrates efferent and reafferent processes and highlights the fact that representational content can be actively mediating behavior while being completely outside the focus of awareness (Roeser and Peck, 2009). Current research suggests non-conscious processes related to self/identity involve repeated associative conditioning of interactions between the body, the environment, and the processes involving exteroception, proprioception, kinesthesia, and interoception (Damasio, 1999; Legrand, 2007; Craig, 2009; Lenggenhager et al., 2009). Exteroception includes the processing of information from all the five senses (vision, audition, olfaction, taste, touch), proprioception—the body in space, kinesthesia—the sense of movement from musculoskeletal feedback, and interoception—the sensation and perception from the internal milieu and visceral organs, including heart rate, digestion, body temperature, and perspiration, among others. The self-specifying sensory-motor convergence that is proposed to contribute to the EES network can be localized to a distributed set of interconnected spinothalamocortical regions [including the periaqueductal gray (PAG) and parabrachial nucleus (PBN), midbrain colliculi, thalamus, hypothalamus, and posterior insular cortex (PIC)], all of which are known to be closely involved in monitoring (orienting and tracking), deciphering, and controlling interoceptive feedback at a non-conscious level (Damasio, 1999; Critchley et al., 2004; Corbetta et al., 2008; Craig, 2008; Northoff and Panksepp, 2008). Self-relevant information first attributes some emotional tone in the ventral posterior nucleus (Vpo) of the thalamus before being somatotopically represented in the posterior and anterior insula (PIC/AIC) (Craig, 2004). The insula somatotopically and hierarchically integrates salient activity and “feelings” progressively in the posterior to anterior direction, where non-conscious homeostatic and motor functions are mapped in the most posterior aspects of the insula and more contextually based relations to one's conscious experience including hedonic, motivational, social, and emotional feelings are represented more anteriorly (Critchley et al., 2004; Craig, 2009).

The EES also involves neural systems involved in preparatory behavior and pre-motor aspects of goal-directed action selection, many overlapping with the DAS. These non-conscious enactive systems are represented in association somatosensory cortices, the pulvinar, intraparietal sulcus (IPS), frontal eye fields (FEF), supplementary motor area (SMA), pre-motor area (PMA), cerebellum, dorsal basal ganglia, superior parietal lobe, and ventral lateral prefrontal cortex (VLPFC) (Decety, 1996; Posner and Rothbart, 2009; Ashby et al., 2010; Gallese and Sinigaglia, 2010). These functionally specified areas are described to be tightly coupled in discrete networks of intentional motor preparation and action (Haggard, 2005; Gallese and Sinigaglia, 2010), early perceptual processing (Brown et al., 2011), search and detection (Corbetta and Shulman, 2002). Based on monkey lesion studies and neuroimaging findings, the VLPFC is implicated as a crucial part of the circuitry in which associations are made between sensory cues and the actions or choices that they specify (Ridderinkhof et al., 2004). Motor learning and procedural aspects of emotional memory formation and expression are conditioned over time, implying automatization and non-conscious enactive representation through repeated forms of stimulus-response behavior. More conscious forms of specialization have been associated with right VLPFC activity (e.g., response inhibition) and it is likely that bilateral VLPFC plays multiple roles in both non-conscious and conscious forms of processing, especially in its integrated role with DLPFC, other motor areas, and the medial temporal lobe (Aron et al., 2004; Dillon and Pizzagalli, 2007).

State-dependent, sustained functional activation of the EES network is likely to represent embodied enaction to support the phenomenological awareness of sensory and mental activity and regulation of attention so mind wandering is less frequent. In a number of studies, experienced meditators (in contrast to non-meditators) were found to have increased BOLD-related activity in areas related to EES during FA/OM or similar meditation practices (Lazar et al., 2000; Ritskes et al., 2003; Brefczynski-Lewis et al., 2007). In a similar population of meditators, morphological increases in gray matter (GM) volume and concentration were found in areas related to EES, including the cerebellum, left SMA, thalamus, caudate, striatum, and parasympathetic control centers of the medulla (Pagnoni and Cekic, 2007; Luders et al., 2009; Vestergaard-Poulsen et al., 2009). Similar findings have been found in long-term practitioners experiencing a peak concentrative state using SPECT to detect cerebral blood flow (CBF) (Newberg et al., 2010). One study found that activation in EES-specific areas (right putamen, PIC) negatively correlated with individual depression scores (Beck Depression inventory), supporting a role for mindfulness in homeostatic and motor function-specific regulation of emotion (Farb et al., 2010). A few other studies have specifically found functional changes in the striatum during meditation. Studies by Lou et al. (1999, 2005) report decreased activity in the caudate during meditation, while others report increases in the caudate-putamen (Lazar et al., 2000; Tang et al., 2009; Baerentsen et al., 2010) and the ventral striatum (Kjaer et al., 2002).

### The experiential phenomenological self (EPS)

Distinctions have been made in phenomenology and the study of consciousness to portray a form of self-specifying experience in which there exists present-centered awareness, and in which the contents of awareness represented at the level of EES are accessible to cognitive systems of modulation, control, and amplification (Block, 1996; Gallagher, 2000; Crick and Koch, 2003; Raffone and Pantani, 2010). The EPS has been referred to as the “*minimal self*” (Gallagher, 2000), “*agentic I”*(James, 1892), the “knower” and “core-self” (Damasio, 2010), all implying the sense of being the immediate subject of experience in the present and to taking a first person perspective (FPP) without reflection or evaluation (Tagini and Raffone, 2010). The EPS is proposed to be a form of higher order conscious, volitional awareness related to exteroceptive and interoceptive experience. This includes the immediate motivational, social, and affective feelings associated with experience, along with top-down attentional control mechanisms found within attentional networks. Top-down processing provides support for exogenous or endogenous forms of engagement, sustained attention, endogenous disengagement capacity, accessibility and storage of information, and executive control mechanisms to regulate encoding, retrieval, and commands for the expression of attention (Dehaene et al., 1994; Block, 1996; Laberge, 2000; Botvinick, 2007; Legrand, 2007; Craig, 2009). The right DLPFC and dorsal ACC have been thought to act in an “executive” capacity for this system, such that they may be responsible for vigilance and alertness—monitoring performance or arousal levels and regulating them accordingly (Posner and Rothbart, 1998; Raz and Buhle, 2006). Along with activity in the temporo-parietal junction (TPJ), these structures have been often associated with capabilities that involve modulating and maintaining response readiness in preparation for an impending stimulus (Raz and Buhle, 2006; Posner and Rothbart, 2009).

According to Craig (2008, 2009), the AIC represents an ultimate “global emotional moment” of the sentient self at one moment of time. The AIC in humans is unique in that it is thought to integrate the higher order, social, emotional, motivational, and cognitive components of subjective feeling states through its strong functional and anatomical connectivity with the ACC, ventral medial pre-frontal cortex (VMPFC), and lateral prefrontal cortex (PFC). This uniquely human aspect of processing high resolution interoceptive information places the AIC in a strong position to handle phenomenological experience related to conscious awareness and likely provides improved self-regulation and autonomic control (Gilbert et al., 2010). Furthermore, AIC activation has been repeatedly shown to be inversely correlated with posteromedial cortex (PMC) [including precuneus, PCC, retrosplenial cortex (RSP)] in functional imaging studies of awareness and task-related attention (Craig, 2009). The PCC and RSP have more often been associated with the NS network, suggesting a mechanism for contrasting momentary awareness with self-reflection.

A number of recent studies investigating neurobiological substrates of mindfulness have indicated very specific changes in the function and structure of the insula and its connectivity with other structures related to experiential self-processing and body awareness. In terms of morphometry, two cross-sectional studies comparing GM morphometry between experienced meditators (8 weeks) and naïve controls have shown greater cortical thickness and GM concentration in the right anterior insula (Lazar et al., 2005; Holzel et al., 2008). A more recent study however (Holzel et al., 2011b) did not find such a change. Long-term vipassana meditation practitioners (>6000 h experience) have shown increased GM concentration in the AIC (Holzel et al., 2008), and functional increases in insular cortex have been found during mindfulness and compassion meditative states (Farb et al., 2007; Lutz et al., 2008a; Manna et al., 2010; Ives-Deliperi et al., 2011). A number of MBI studies have demonstrated the functional role of insular cortex in states associated specifically with the EPS. For example, meditators using an experiential FPP (in comparison to an evaluative focus) toward a group of valenced trait descriptive words resulted in a pronounced shift away from midline cortices toward a right lateralized network comprised of the VLPFC and DLPFC. In addition, increases in BOLD activity were found specifically in right AIC, somatosensory cortex (SII) and the IPL (in comparison to control subjects) (Farb et al., 2007). Strong functional coupling was found between the right PIC and mPFC in novices that was not present in the meditators, who showed more significant connectivity between right PIC and DLPFC. This suggests a fundamental neural dissociation between critical structures related to interoception and evaluation that are habitually integrated, but dissociated through meditation training. Manna et al. (2010) found the left AIC cluster in OM practice to be positively correlated with areas related to executive monitoring and attention in the left hemisphere (anterior PFC and sPL, STG). Further studies of meditation have found similar increases in right AIC activity coupled with decreases in posterior parietal cortex and cortical midline structures (Creswell et al., 2007; Holzel et al., 2007; Farb et al., 2010), while others have found increased functional connectivity between areas also implicated in present-centered episodic awareness (DMPFC) and sensory areas (SI, SII) (Kilpatrick et al., 2011). Andrews-Hanna et al. (2010) also demonstrated that a FPP episodic judgment task concerning one's present situation activated a distinct network referred to as the dorsomedial PFC (DMPFC) subsystem, including the TPJ, lateral temporal cortex (LTC), and temporal pole (TP). There appears to be a role for this subsystem with EPS circuitry and prosocial behavior. Another study found that interoceptive awareness (in contrast to exteroceptive awareness) in individuals trained in MBSR recruited decreased DMPFC activity and negative functional connectivity with PIC (Farb et al., 2012), suggesting a critical role for DMPFC in modulating primary interoceptive areas. The precuneus is also involved in contexts involving a FPP, as well as visuo-spatial imagery, execution and preparation of motor behavior, episodic memory retrieval, and agency (Cavanna and Trimble, 2006). Furthermore, the precuneus and surrounding PMC are amongst the brain structures displaying the highest resting metabolic rates (i.e., 35% more glucose consumption than any other brain area), suggesting a tonic role in self-processing. Interestingly, the precuneus is characterized by transient decreases in tonic activity during engagement in NS-processing or in default mode forms of rest (Cavanna and Trimble, 2006). The precuneus has been found to be activated during FA > Rest, and in OM > Rest, suggesting it play a key role in self-induced transitions between meditative and discursive rest states (Manna et al., 2010). These data further support that mindfulness training facilitates volitional flexibility for switching between the self as subject and object. Moreover, future research will have to clarify how EPS networks interact with executive attention and primary interoceptive brain areas through context-specific modalities of present-centered awareness.

In the context of pain, there has been mixed results in terms of insular activity. For example one study of experienced meditators (39–1820 weeks of meditation practice), in comparison to non-meditators, showed decreased left PIC and SII activation in the anticipation period that continued through the experience of pain (Brown and Jones, 2010). In contrast, two studies showed no difference during anticipation and stronger activation in pain-related areas (mid-cingulate, insula, SII, and thalamus) during experience of pain and while maintaining a “mindful state” (Grant et al., 2010a; Gard et al., 2012). Greater bilateral activation in the PIC, along with somatosensory areas corresponding to the nose and throat, was found in meditators given brief training experience (4 × 20 min sessions) while practicing FA meditation (Zeidan et al., 2011). Zeidan et al. (2011) also showed decreased activity in the PIC and somatosensory areas corresponding to the site of pain stimulus while meditating in the context of noxious stimuli. Interestingly, right AIC and dACC negatively correlated with pain intensity, while OFC activity negatively correlated with pain unpleasantness (Zeidan et al., 2011). These studies of acute pain suggest meditators modulate primary interoceptive pain processing (in comparison to controls) during pain experience and affective dimensions of pain processing are diminished.

While most meditation practitioners often report having enhanced awareness of body sensations, there is a paucity of good evidence that they actually do so (Nielsen and Kaszniak, 2006; Khalsa et al., 2008). For example, one study in particular compared two groups of advanced meditators (Tibetan and Kundalini practitioners; >15 years formal practice) against a control group of non-meditators at two different time points on a common interoceptive heart-beat detection task and pulse detection task (Khalsa et al., 2008). During pulse detection, participants took their non-dominant wrist pulse and were required to judge whether a train of exteroceptive stimuli (800-Hz, 50-ms tones) were simultaneous or non-simultaneous with pulse sensations. On neither task did the meditators perform better in terms of perceptual accuracy. Given the sufficient power and control of the study, it is possible that awareness of heartbeat synchrony may not be a good general index of the type of awareness that is cultivated by mindfulness training. Future studies will have to examine more subtle forms of interoceptive awareness that appear to recruit the left AIC, for example. Other sensory modalities that are targets of the contemplative practice will have to be explored as well. For example, tai chi practitioners have shown to have superior tactile acuity compared to non-practitioners (Kerr et al., 2008).

### The evaluative narrative self (NS)

The NS is an evaluative, reflective form of identification that follows experience in narrative form, described in some contexts as the “Me-self” or “material me,” the sum total of all a person can call their own across physical, social, or psychological domains: “That looks like me,” “Am I a nice guy?,” “I feel anxious” (Brewer, 1993; Craig, 2004; Damasio, 2010; Tagini and Raffone, 2010). The NS is also reflected in metacognitive knowledge, access to knowledge people have about their cognitive abilities (“I have a bad memory”), about cognitive strategies (“to remember a phone number I should rehearse it”), about tasks (“categorized items are easier to recall”), etc. (Flavell, 1979). There may very well be distinct substrates across each domain as there is likely distinct forms of “me-self” in as many social relationships as there are individuals who recognize each one uniquely. The common feature in the NS is an awareness of a specific object, “me” to which identification with, and evaluation of, specific characteristics are made. The recurrent feedback of self-identification embedded in cognitive self-schemas perpetuates a stable sense of self. This is illustrated in the dysfunctional representation in Figure 1.

**Figure 3:**
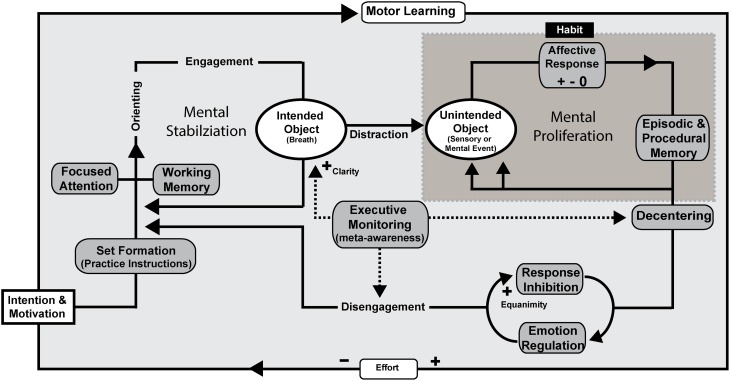
**Mindfulness process model—concentrative practice.** FA meditation using the breath as the object of focus is illustrated as an example, but the processes are proposed to be the same across concentration styles of practice. Intention is formed along with motivation to practice before an executive “set” is created. Executive set is supported by the working memory system in order to implement and maintain practice instructions. Focused attentional networks are recruited and sustained along with support by component mechanisms including executive monitoring, emotion regulation, and response inhibition. Unintended objects of distraction can include any stimulus available to extero- or interoceptive sensory and mental processes. Affective responses to unintended objects can have a positive, negative, or neutral valence and are likely to proliferate endlessly unless awareness and de-centering promote response inhibition and disengagement. Motor learning provides a framework for automatization and mindfulness skill development. Effortful control is reduced through continued practice. Through practice, awareness itself becomes the object of attention in meta-awareness as the meta-function is acquired as a skill. Clarity, as a form of phenomenal intensity during practice increases as does equanimity, which refers to impartiality reducing later attentional and emotional stages of strategic processing that could potentially involve prolonged sympathetic arousal, cognitive elaboration, or ruminative qualities.

The NS is mediated by the HCMS—a network of cortical midline structures sometimes referred to as the “E-network” due its evaluative nature (Northoff and Bermpohl, 2004; Legrand, 2007; Schmitz and Johnson, 2007; Legrand and Ruby, 2009). This network includes the VMPFC, pre- and subgenual anterior cingulate cortex (pACC; sgACC), medial parietal cortex (MPC), PCC, and RSP (see Figure 3). Because of the dense reciprocal projections with anterior thalamus and hippocampus, the RSP and MPC both aid in the repeated construction of identity in time and space through moment-to-moment episodic memory formation (Peters et al., 2009; Spreng et al., 2009). Through interactions between PMC and subcortical limbic structures (e.g., hippocampus, amygdala), there is a progressive accumulation of an “autobiographical self,” a set of memories that make up the individual's unique past, current state, and expected future (Zelazo et al., 2007; Spreng et al., 2009). The VMPFC is heavily interconnected with the amygdala and ventral striatal pallidal complex (including nucleus accumbens), supporting the representation of affective and motivational states through a gradient of non-conscious and conscious forms of affective appraisal. Through an interaction with the sgACC, there is evidence suggesting a mechanism for appraising the value of stimuli with respect to current goals and decisions, while some data suggest a role in modulating emotion and disengagement through executive control mechanisms (Drevets et al., 2008; Roy et al., 2012). Increased NS activity during goal-directed tasks is often associated with excessive and repetitive elaboration or rumination, decreasing efficiency of the attentional system and capacity of information processing in general, further supporting the claim that increased NS processing can lead to negative health outcomes (Brewer, 1993; Mogg et al., 1995; Rimes and Watkins, 2005; Grimm et al., 2009).

A number of studies investigating forms of psychopathology have reported decreased task-induced deactivation of the NS network, suggesting increased mind-wandering and self-reflection during ongoing task demands (see Broyd et al., 2009). A few studies on states of meditation have reported decreased task-related mind wandering and increased task-related deactivation of NS activity (Farb et al., 2007; Ott et al., 2010b; Brewer et al., 2011). Some studies of meditative states have shown a similar effect in the context of distraction, suggesting increased engagement, and decreased mind-wandering and reactivity to distractions (Anderson et al., 2007; Brefczynski-Lewis et al., 2007; Pagnoni et al., 2008; Grant and Rainville, 2009; Farb et al., 2010; Manna et al., 2010). Similarly, increased functional connectivity has been found within areas specific to the NS network (e.g., mPFC) and regions of primary interoceptive awareness of meditators (~1000 h practice) (Jang et al., 2010; Brewer et al., 2011), while studies using the PCC as a seed found increased connectivity with dorsal ACC and DLPFC at rest and in the dorsal ACC during meditation (Brewer et al., 2011). These correlations between anticorrelated networks may be interpreted as evidence for increased integration and volitional control in recruiting networks when contextually appropriate. For example, Josipovic et al. (2011) demonstrate that such correlations are stronger during FA meditation, but smaller during OM forms of practice.

Across a number of different MBI studies (Lazar et al., 2005; Pagnoni and Cekic, 2007; Holzel et al., 2008, 2010, 2011b; Luders et al., 2009; Vestergaard-Poulsen et al., 2009; Grant et al., 2010a), increases in GM volume and density (in comparison to non-meditating controls) have been found using voxel-based morphometry (VBM) in hippocampus and PCC. Such changes further suggest meditation training increases efficiency of memory networks related to the NS, forseeably providing more control over its expression. Increased GM concentration in the right hippocampus has also been attributed to training in arousal regulation, suggesting another mechanism for regulating the self toward more adaptive trajectories of self-experience. The S-ART framework suggests mindfulness critically involves working memory, efficiency of memory encoding, retrieval, and extinction processes, all aspects of hippocampal and parahippocampal activity. These data also suggest an enhanced ability for pattern separation (transforming similar representations into highly dissimilar, non-overlapping representations), inhibition of mental contents or behavioral schemas that are unrelated to the focal goal in working memory, and facilitation of attentional disengagement from distractor stimuli.

### An integrative fronto-parietal control network for S-ART

Exploration of the current literature suggests that an integrative network supported by mindfulness may improve efficiency and guide changes in self-specific, affect-biased attention by integrating information from the three self-specific networks. The fronto-parietal control system (FPCS) [rostral frontopolar PFC (FPC), right VLPFC, DMPFC, dorsal ACC, DLPFC, AIC, lateral cerebellum, and anterior inferior parietal lobe (aIPL)] is uniquely situated to integrate information coming from the other three systems and facilitate global reorganization or plasticity amongst the networks. The aIPL and DLPFC have been implicated in working memory, and are thought to contribute to regulating attention within this network. As described earlier, VLPFC shares conscious and non-conscious roles in recollecting mnemonic associations and whether there is conflict in the actions/choices being made in reference to the present context (Ridderinkhof et al., 2004). The VLPFC has also shown to be active during such effortful and controlled processing including inhibition of set-switching, and inhibitory control (e.g., as in go-nogo tasks) (Posner and Petersen, 1990). The FPC has also been implicated in top-down monitoring and described by some as the “supervisory attentional gateway” (SAG) controlling stimulus-independent and stimulus-oriented cognition (Burgess et al., 2007). Furthermore, FPC has been associated with coordinating information processing between multiple brain regions (see Vincent et al., 2008). It is these substrates that support introspective meta-monitoring and the relational binding necessary for self-awareness and self-regulation.

The FPCS has some functional overlap with the EPS network (including DLPFC, DMPFC, AIC), suggesting present-centered awareness is critical for self-regulation and targeting attentional bias. The FPCS has been emphasized by Vincent et al. (2008) and others (Seeley et al., 2007; Liao et al., 2010; Deshpande et al., 2011) to integrate task positive and task negative networks through executive control and salience processing. There is now some research to suggest that inadequate integration between the self-specifying networks and the NS network may be a significant contributor to emotion dysregulation (Bressler and Menon, 2010), and affective biased attention (Todd et al., 2012). For example, Bressler and Menon (2010) suggest that salience processing by AIC and dorsal ACC play a causal role in switching between task positive and task negative networks (Bressler and Menon, 2010). Todd et al. (2012) proposes that affective biased attention operates before conscious cognitive reappraisal or suppression strategies, suggesting poor feedback from the EES network and subsequent prioritization of overlearned habitual responses. It may be the case that the development of meta-awareness facilitates the role of FPCS in integration and increased efficiency of the networks for S-ART. Thus, through mindfulness training, cognitive and emotional resources are proposed to be utilized more effectively and in an adaptive fashion.

The three most widely cited brain areas of activity and gross morphological change during and in response to both FA and OM meditation training has been the DLPFC, the ACC, and the insula [see Lutz et al. (2007); Chiesa and Serretti (2010); Rubia (2009); Holzel et al. (2011a) for review], suggesting FPCN and EPS-related processing is heavily influenced through training. Across nine different MBI studies (Lazar et al., 2005; Pagnoni and Cekic, 2007; Holzel et al., 2008, 2010, 2011b; Luders et al., 2009; Vestergaard-Poulsen et al., 2009; Grant et al., 2010a; Manna et al., 2010), differences in GM volume and density (in comparison to non-meditating controls) have been found using MRI in the dorsal and rostral ACC, suggesting a prominent role for the FPCN. Tang et al. (2007, 2009, 2010) show that as little as five days (20 min/day) of Integrated Body Mind Training (IBMT), involving components of FA and OM produces greater activation in the rACC during rest. From as little as 11 h (over 1 month) of IBMT, increases in structural connectivity were found indicated by increased fractional anisotropy, an MRI diffusion tensor imaging index indicating the integrity and efficiency of white matter connecting ACC to other cortical and subcortical structures. One study investigating acute pain in experienced meditators found that mPFC/rACC activation was negatively correlated with pain unpleasantness ratings in a non-meditative state (Brown and Jones, 2010). In the control group, the opposite correlation was found with overall lower activity, suggesting less attentional control during experience of acute pain. Two studies of samatha-vipassana practitioners (5000 to >10,000 h practice) demonstrated increased BOLD activation (compared with non-meditators and within the meditation group) in rostral ACC and DMPFC during FA meditation (Holzel et al., 2007; Manna et al., 2010). Manna et al. (2010) also found meditation-specific increases in precuneus, bilateral IFG, and right parahippocampal gyrus during OM > Rest condition. Interestingly, the same study found that during the FA meditation, the right IFG and left PIC positively correlated with meditation expertise (number of hours of practice) (Manna et al., 2010). Given the overlap across networks, these data further support a role for the FPCN in improving network interactions in advanced meditators. Many of these activations show dominance in the left hemisphere (in comparison to rest), which may reflect increased positive dispositions (Davidson and Irwin, 1999) and increased meta-awareness (Tagini and Raffone, 2010). Furthermore, advanced meditators show resistance to age-related decline in FPCN and self-specifying brain areas. For example, one study of Vipassana practitioners (>2500 h of practice) found increased cortical thickness in AIC, DLPFC, anterior PFC, and sensory cortices (Lazar et al., 2005). Normal age-related decline in GM volume and in attentional performance was present in controls but not in meditators, suggesting that meditation slows the natural progression of cortical thinning over time.

## Mindfulness-based mental training: methods for adaptive self-functioning and integrating self-specific networks supporting S-ART

**Figure 4:**
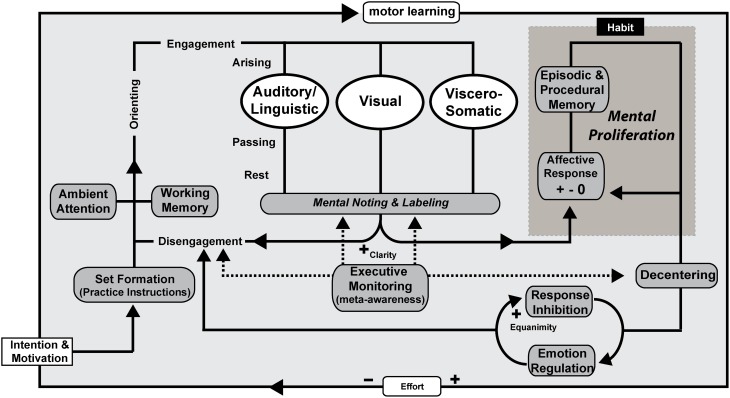
**Mindfulness process model—open monitoring receptive practice.** OM meditation with no object of focus (ambient attention) is illustrated as an example, but the processes are proposed to be the same across receptive styles of practice. Intention is formed along with motivation to practice before an executive “set” is created. The working memory system helps to maintain motivation and practice instructions. Ambient (diffuse) attentional networks are recruited and sustained along with support by component mechanisms including executive monitoring, emotion regulation, and response inhibition. Mental noting and labeling of stimuli arising, passing, and absent in phenomenal conscious awareness is a form of emotion regulation and contributes to extinction and reconsolidation of maladaptive procedural and declarative memories that represent sensory-affective-motor scripts and schemas. Affective responses may arise in response to an object of attention with a positive, negative, or neutral valence and are likely to proliferate unless awareness and de-centering promote response inhibition and disengagement. Over time and continued practice, effortful control is reduced and awareness itself becomes the object of attention as meta-awareness is cultivated as a skill. Clarity and Equanimity increases through practice.

**Figure 5:**
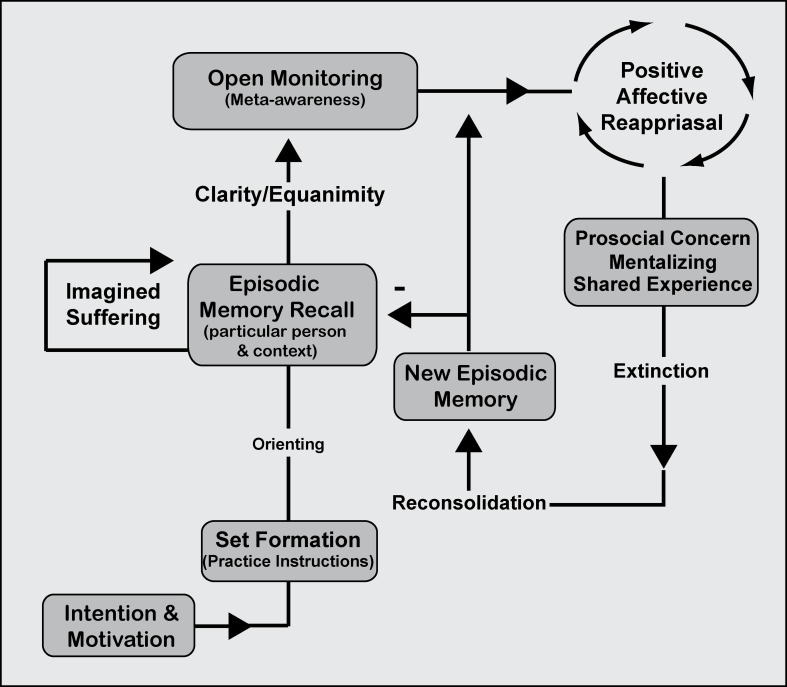
**Mindfulness process model.** Ethical-enhancement (EE) practice. Intention is formed along with motivation to practice before an executive “set” is created within the working memory system in order to implement and maintain practice instructions. Focused attentional networks are recruited for orienting toward sustained episodic memory recall involving mentalizing and shared experience. OM practice is described to supplement EE practice facilitating awareness of any modality of experience that arises. The processes are proposed to be the same across ethical styles of practice that require imagined suffering or a particular declarative memory [of someone or something]. Mentalizing one's own or others' suffering continues recurrently as part of sustained episodic memory recall. With clarity and equanimity for affective reactivity, OM practice allows the practitioner to remain mindfully aware of difficult emotions while the declarative (episodic) memory is positively reappraised. Negative associations are extinguished and reconsolidated into more adaptive or positive memories using prosocial/empathic concern for the object of meditation. Continuous reappraisal along with prosocial concern is thought to enhance exposure, extinction, and reconsolidation processes such that new episodic memories are laid down and inhibit older maladaptive forms of the memory.

There is now evidence to suggest that mindfulness practice can modulate and produce enduring neuroplastic changes, including gross morphological changes, across the self-specific networks, and an integrative FPCN supporting S-ART. The extant data support a strong emphasis on activity of the self-specifying networks and decreased NS activity during baseline and goal-directed activity after mindfulness training. Additionally, increased activity is commonly found in the EPS network and FPCN during meditation practice. Yet, multivariate network types of analyses have not demonstrated conclusively how all the networks interact in the context of meditation training and practice, during basal states of inactivity, and during goal-directed tasks. Given the role of the FPCN in executive control and in integrating information between task positive and task negative networks, mindfulness may effectively facilitate context-appropriate switching between anticorrelated networks, rather than simply increase the functional activity of experiental self-processing or suppression of NS. The evidence suggests state-dependent activation and integration of task positive and task negative networks support reduction of self-specific biases and may contribute to a sustainable healthy mind; however, such hypotheses need to be further tested. Here we explicitly describe how three types of mindfulness-based meditation practices function by illustrating the putative neurocognitive mechanisms in conceptual process models (see Figures 3–5) and how each component mechanism may map onto the self-specific networks identified above to support S-ART.

### Focused attention (FA) concentration practice

FA practice involves sustained attention on a specific mental or sensory object: a repeated sound or mantra, an imagined or physical image, or specific viscerosomatic sensations. The object of focus can be anything, but the method described by the Satipatthāna Sutta identifies a naturally occurring breath focus. In fact, breath is a particularly apt foundation because it integrates conscious awareness with ongoing, dynamic viscerosomatic function. The goal of FA practice is to stabilize the mind from distraction, torpor, and hyperexcitability, all of which are predicted to be negatively correlated with practice. Effort is hypothesized to be inversely correlated with practice duration, providing a neurobiological mechanism for advancement of the practitioner and resulting in decreased allocation of explicit cognitive resources over time. A process model for FA is illustrated in Figure 3.

FA practice is described here to involve an underlying framework of motor learning that functions to strengthen non-conscious, associative memory processes underlying the EES network (and subcortical analogs), and which support the conscious, explicit processes recruited during practice. Intention, a critical component of mindfulness in previous conceptual models (see Shapiro et al., 2006), is described to be critical for motivating the practitioner to begin/sustain practice and activating EES networks that may be the initial steps for extinguishing maladaptive habitual perceptual-motor action tendencies. Intention and motivation may also target attentional tuning and affective control settings which contribute to affect-biased attention (Todd et al., 2012). The instructions for practice form an executive “set” that is created and sustained by working memory processes, while attentional processes operate to focus and sustain concentration on the intended object. Alerting, orienting, engagement, and disengagement all involve discrete networks (Posner and Petersen, 1990) which complement one another in their contributions to the FA practice. An executive monitoring function is proposed to provide feedback when the goal state of concentration on the particular object has shifted and to support the attentional processes related to the return of focus. Meta-awareness is described to arise as a highly developed form of executive monitor that allows the practitioner to have awareness as the object of attention while simultaneously maintaining awareness in its natural state, whichever object of attention it may be resting upon. Sensory clarity is presumed to increase in proportion to the strengthening of meta-awareness. Given the perspective shift one must learn through development of meta-awareness, decentering is described as a psychological process that may be a mechanism to support disengagement processes and sensory clarity.

Equanimity and clarity are both processes that support concentrative practice and are proposed to increase in terms of efficiency and phenomenal quality through time and practice. Similar to the Buddhist description (see Section “Defining Meditation and Mindfulness from the Historical Perspective”), equanimity refers to response inhibition and emotion regulation processes that reduce later attentional and emotional stages of strategic processing potentially involving prolonged sympathetic arousal, cognitive elaboration, or ruminative qualities which may arise in response to an object of distraction. Increased clarity of experience is proposed to operate as one develops an increased phenomenal awareness of the breath and objects of distraction arise and disappear, without necessarily affecting the cognitive access of the contents of conscious experience.

### Open monitoring (OM) receptive practice

Figure 4 illustrates a process model for the receptive practice, OM, which is described in detail elsewhere. Novices begin by actively monitoring and labeling external (exteroceptive) or internal (interoceptive) domains of the five senses, including viscerosomatic states and inner speech. The specific sensory modalities provide the scaffolding for meta-awareness to develop in each domain and in relation to self-related processing. There is an explicit distinction between engagement and disengagement with an object early on in the practice. Provided the framework of motor learning, advancement of the practitioner is likely to automatize aspects of attentional processing and improve efficiency of engagement and disengagement processes, thereby reducing bias associated with orienting of attention. For example, when distraction arises, and the practitioner gets “stuck” in thought or emotion, a number of mechanisms supported by mindfulness-based practices may facilitate disengagement. The practitioner is encouraged to continually rest in awareness of intero- and exteroceptive stimuli, and any cognitive or emotional states that may arise. Through training, the practitioner can learn to note and sustain attention on the arising, passing, or absence of each modality of experience—naturally reducing the frequency of cognitive evaluation or rumination. Eventually, through training, the process of mental noting becomes effortless and non-reactive, qualities that describe the development of equanimity. At this point, efficiency of the attentional system has improved, resulting in decreased allotment of attentional resources toward any particular feeling, image, or thought. Cognitive emotion regulation strategies like reappraisal or suppression may be more prevalent in novice practitioners dealing with distraction, but less so in advanced practitioners as attentional systems are strengthened and qualities of equanimity arise. One may hypothesize that the changes from novice to advanced practitioner may be due to the development of psychological processes like non-attachment, de-centering, or from a non-conscious shift in affect-biased attention and the development of meta-awareness. Whichever the mechanism, maladaptive scripts and schemas related to the self are proposed to be extinguished and reconsolidated through a combination of FA and OM practice.

Similar meditation practices instruct the practitioner to maintain a diffuse form of attention or ambient attentional focus such as “Shikantaza” or “just sitting” in Japanese Soto Zen (Austin, 2006); “choiceless awareness” by Krishnamurti (1969), insight meditation from Theravada (Pali: *Vipassana*) (Lutz et al., 2007), and “absolute rest,” by other western Buddhist teachers (Young, 2010). Vipassana, has also been translated as “removing the veil of ignorance” (Gunaratana, 2002), such that this stage of meditation practice explicitly aims at providing insight into one's self-related processes. In advanced practices, the practitioner can reside in a non-dual level of awareness, referred to by the Buddhist Abhidharma scholars as a clear understanding of no distinction between knowing subject and perceived or known object (Dunne, 2011). Some Buddhist traditions have referred to this non-dual practice as “Open Presence” (Thangru and Johnson, 2004; Lutz et al., 2007), “open awareness,” “rig.pa” in Tibetan forms of Dzogchen (Rinpoche et al., 2006), or “phyag.chen” in Mahamudra (Thangru and Johnson, 2004). Non-dual practice involves the decreased reliance on engagement and disengagement processes, mental noting or labeling techniques. Such changes in the practice are likely to affect the relationship the practitioner has with his/her own thoughts, so that cognitive structures related to ones' thought and emotion that represent reality are “de-fused” from what is considered reality itself (Hayes et al., 2003; Dunne, 2011).

### Ethical enhancement (EE) practices

The diminishing of grasping, aversion, and delusion that arises in insight meditation practice naturally leads the practitioner to greater loving-kindness toward oneself and others (Salzberg, 2011). The cultivation of loving-kindness (Pali; *metta*), or forgiveness can also have very explicit instructions for visualization and is based on the premise that all beings want to be happy. The following excerpt comes from one of the original teachings of loving-kindness practice.

Even as a mother protects with her life

Her child, her only child,

So with a boundless heart

Should one cherish all living beings

(Karaniya metta sutta, 1.8)

Contemporary instruction for the cultivation of loving-kindness can be found in the works of Sharon Salzberg (Salzberg and Bush, 1999) and by Matthieu Ricard (Ricard, 2003), but such practices are based on the method found in Buddhaghosa's 5th century text, the *Path to Purification* (Buddhaghosa, 1991). This traditional approach is characterized by progressive cultivation of loving-kindness toward oneself, a good friend, a neutral person, a difficult person, all four of the above equally, and then gradually the entire universe. The loving-kindness is cultivated in reference to a specific declarative (episodic) memory of each particular individual or event. The practitioner is encouraged to avoid choosing someone to whom they are sexually attracted or who is dead (Ricard, 2003). For a “neutral” person, the practitioner is encouraged to choose someone they may come into contact with every day, but who does not give rise to strong positive nor strong negative emotions. For a “difficult” person, the practitioner is encouraged to choose an enemy, or someone strongly disliked. Furthermore, the practitioner is encouraged to break down the barriers between self and other by practicing loving-kindness repeatedly, achieving impartiality toward the four persons, including him/herself, the close friend, the neutral person, and the hostile person. The EE practice is illustrated in Figure 5.

Compassion practice has also been described as an EE style of practice; however, without the simultaneous cultivation of open presence along with the generation of compassion, the practice has been described to be unsuccessful (see Lutz et al., 2007). Compassion practice is also described as an enactive, emergent process of factors in the attentional and affective domains, the intentional and insight domains, and the embodied and engaged domains of subjective experience (Halifax, 2012). Similar to the metta practice described above is Tonglen, a Tibetan Buddhist term for the meditation technique translated as “giving and taking” or “sending and receiving” in which the practitioner visualizes taking onto oneself the suffering of others on the in-breath, and on the out-breath giving happiness, joy, and kindness (Lama Xiv et al., 2001; Lutz et al., 2007).

## Six component neurocognitive mechanisms of mindfulness

As illustrated, mindfulness is not a unitary construct and the process models are an attempt to illustrate the cognitive and psychological processes that support the meditation practices. These component processes provide the proposed mechanisms by which mindfulness works to increase S-ART—the framework for reducing self-specific biases and sustaining a healthy mind. The S-ART framework includes six component supportive mechanisms underlying the practice and cultivation of mindfulness as a state and trait: (1) intention and motivation; (2) attention regulation; (3) emotion regulation; (4) memory extinction and reconsolidation; (5) prosociality; (6) non-attachment and de-centering. Here we describe the neurobiological substrates of each mechanism and provide support from the extant contemplative science literature.

### Intention and motivation

As originally proposed by Shapiro and colleagues (2006), intention is one of the fundamental building blocks out of which an array of neurocognitive mechanisms may emerge to effectively cultivate mindfulness. Intention and motivation are based on inter-related aspects of affective style and biological disposition, driving goal-directed behavior (Davidson and Irwin, 1999; Ryan and Deci, 2000). External and internal motivation have been distinguished as two unique classes of motivation. Although both classes refer to incentives of reward, external motivation arises from the desire to approach (avoid) an externally imposed reward (punishment); while internal motivation arises from a more automatic, internally driven desire to obtain reward, or satisfy one's needs (Davidson et al., 2000; Ochsner and Gross, 2005; Rothman et al., 2011). People are clearly motivated to practice by very unique factors with highly varied experiences, incentives, and consequences; however, through advancement of practice, motivation is proposed here to become more internally driven (i.e., suggesting increased control) and less focused on outcome. Intention refers more specifically to a purposive plan of action that is selected and the timing selected for such action (Krieghoff et al., 2011).

Motivation and intention are described as a feedback system for self-schema maintenance. As in any feedback system, sensory input is continually compared to a reference, and discrepancy from the reference feeds back into frontal goal-oriented networks and eventually to pre-motor-action networks modulating functional output. If the reference is some ideal that is unrealistic or if the interpretation of incoming signals related to the self are continually negative in valence, then there is an error signal of which the rate of discrepancy reduction may never progress, perpetuating negative affect (Carver and Scheier, 2011). Negatively biased cognitions and/or distorted perceptions that involve disinterest, disapproval, or rejection have been shown to result in nonsocial, isolating behaviors (including avoidance and withdrawal), impaired self-regulation, and psychopathology; conversely, current data suggest that approach behavior including more engaging perceptions involving interest, approval, or acceptance typically lead to healthier outcomes (Waikar and Craske, 1997; Sin and Lyubomirsky, 2009). Thus, internally driven motivation to engage with experience without craving or aversion (i.e., with equanimity) is suggested to increase self-regulation.

Davidson and Irwin (1999) suggest there are two fundamental resting patterns of neural activity for motivational systems of approach and avoidance. Left anterior PFC activation has been associated with positive affect and increased right-sided anterior activation with negative affect, suggesting that individual differences in asymmetric activation of these brain regions can be associated with dispositional differences in a biological set point for motivation and which may contribute to the overall outcomes of mindfulness training. Davidson and Irwin (1999) further delineates functional differences between ventromedial and dorsolateral sectors of PFC, suggesting that activation of the former represent immediate valenced goal states along with activation of the ventral striatopallidum [incl. nucleus accumbens (nAcc)], while the latter area DLPFC represent valenced goal states in the absence of immediately present incentives. In other words, DLPFC may contribute to a sustained form of motivation that remains in working memory during the meditation practice. Furthermore, animal and human research suggest that activity in the PFC can modulate the ventral striatopallidum and specifically nAcc activity in a top-down manner, postulating a mechanism for tracking internally driven, approach forms of motivation (Heller et al., 2009) as well as regulating aspects of craving associated with more phasic nAcc activity (Robbins and Everitt, 1996). More recent data suggests that decreased ventrolateral PFC (VLPFC) activity may also be evidence for increased approach behavior and expression of positive affect (Light et al., 2011). The contributions of these substrates to habits and automatization are typically described to be hard-wired, but through FA/OM and ethical based practices, it is proposed that extinction and reconsolidation mechanisms (see Section “Extinction and reconsolidation”) can shift biological set-points and modify pathological scripts and schemas into more adaptive trajectories.

There has yet to be extensive investigation of motivation, intention, and reward in meditation and mindfulness research; however there is some PET data demonstrating increased endogenous dopamine release in the ventral striatum during Yoga Nidra meditation (Kjaer et al., 2002), a finding accompanied by a reported “decrease desire for action.” Expression of loving-kindness, a sense of connectedness, and feelings of trust and cooperation have all shown to produce increased activity in the ventral striatum (Cialdini et al., 1997; Bora et al., 2009). Significant increased activations in rostral ACC and OFC have been found in the majority of non-guided meditation studies, suggesting effects related to attentional focus, but also likely related to tonic intentional activity.

### Attention regulation

Volitional shifting of conscious awareness between objects of attention in a serial and/or parallel fashion is suggested to be a critical process for effectively managing or altering one's responses and impulses. This executive, volitional management of attention can explicitly control where and when attention shifts in top-down fashion. Regulation is also believed to occur from a bottom-up direction, such that cognitive resources are conserved and non-conscious afferent-efferent systems interact with the environment in an efficient, adaptive feedback loop with top-down systems. Concentration forms of practice are proposed to increase the efficiency of the attentional system, including the dissociable, but interrelated sub-components of attentional processing. The subcomponents are identified in the concentrative practice process model (Figure 3) and include alerting and orienting toward an intended object of interest, engaging with the object, sustaining attentional focus, executive monitoring and detecting distraction, disengaging from the source of distraction, and re-engaging on the intended object (Posner and Petersen, 1990; Corbetta and Shulman, 2002; Raz and Buhle, 2006; Posner and Rothbart, 2009).

Efficiency and stability are also measured by sustained attention (i.e., vigilance and alertness), referring to the capacity to detect unpredictable stimuli over prolonged periods of time (Posner and Rothbart, 1998). Deep engagement, vivid absorption, or concentration power, is an embodied state of awareness in which no other sensory or internally generated input can arise beyond a perceptual threshold (Rahula, 1974; Bodhi, 1999). Full absorption in an object with focal awareness is critical for stabilizing the mind during FA meditation; however, it can be maladaptive, such that inhibitory processes can prevent pertinent sensory information from arising to conscious awareness, decreasing available cognitive resources for ongoing task demands, and potentially leading to an overwhelming sensation and maintenance of emotional reactivity related to the object of focus (Mogg and Bradley, 1998). Regulatory efficiency and stability are also measured under various conditions of perceptual load or conflicting contexts (e.g., Stroop phenomena), through continued performance with fewer distractions, through the rapid disengagement from an object of focus to another intended object in rapid succession (e.g., attentional blink paradigm, dotprobe task), and through an ability to shift back and forth between local and global features (e.g., perceptual rivalry task). The neural substrates for these attentional processes are described through S-ART networks. For example, preparatory forms of attention are described through EES circuitry, while the substrates for FA and executive monitoring are described through EPS and the FPCS. It is likely that distinct circuitry may also be identified for arising, passing, and restful states of concentration for each modality of focus.

Although people generally claim to have full conscious awareness of their environment when their focal attention is directed outward or toward their own internal thoughts, attention research shows that this is quite false. In fact, it is very likely that particular information from the environment is filtered out unless reaching some perceptual or semantically meaningful threshold before it can gain access to conscious awareness. At a very basic perceptual level, our focal awareness is very small relative to the rest of the context around that focus, such that we experience a phenomenon called “change blindness” when cues such as motion, that normally lead to a shift of attention, are suppressed and major changes across sensory or semantic modalities in the remainder of the environment are not reported (Simons and Levin, 1997; Posner and Rothbart, 2009). There is ample evidence that early stages of attentional processing and fleeting perceptual traces of sensory information exist before there is conscious awareness (Pessoa, 2005). Receptive forms of meditation (i.e., OM) facilitate more diffuse or ambient attentional mechanisms and consequently, phenomenological awareness of the objects in conscious experience is likely enhanced, without necessarily increasing cognitive access [see (Davis and Thompson, forthcoming)]. Thus, reportability for states of interoception and exteroception may not necessarily be improved, while more subtle, non-conscious processes supported through EES may improve in efficiency. These forms of early, non-conscious processing can be measured in pre-categorical, high capacity sensory memory stores (Sperling, 1960; Chrousos and Gold, 1992), perceptual rivalry, or affect-biased attention (Attar et al., 2010; Todd et al., 2012). Future research in the contemplative sciences will likely reveal more subtle changes in non-conscious states of early stages of perceptual and attentional processing. A recent pilot study used an iconic memory task, in which an array of eight letters was displayed for 30 ms and a target letter was to be identified up to 1000 ms after the trace was removed. The study found dispositional mindfulness, non-attachment, and compassion in advanced meditators was not associated with accuracy, but positively correlated with the subjects' ability to identify a letter adjacent to the correct target and in the larger array. It is proposed, therefore, that such dispotional factors leads to increased diffuse attention to the periphery without improving iconic perceptual memory traces to the target focus of an 8-item array (Fischer et al., 2012).

Meta-awareness points to the possibility of taking awareness itself as an object of attention and can be disambiguated from sensory-conceptual domains of self-experience especially evident in the NS (Lutz et al., 2007). Meta-awareness contributes the critical mnemonic aspect of mindful awareness responsible for monitoring the meditative state so that one may “see and know” when they have lost the focus of attention on the object, or emotion has become reactive or ruminative. Likewise, the same meta-awareness serves to determine whether there is dullness or too much excitation during the practice and therefore, contributes to the phenomenal intensity or clarity in which the meditation is experienced (Lutz et al., 2007). Without such a highly developed sense of self-awareness, the contents of subjective experience are continually entangled with the patterns of conditioned and consolidated schemas that dictate behavior. Husserl refers to this abstracting from all objects as “epoche,” or bracketing objects of conscious experience in order to reflect on the contents within it (Varela et al., 1991). Meta-awareness may differ from the EPS only in its relation to the world, from which one is able to study the intentional contents of the mind in a transcendent, non-conceptual way.

Recent studies have investigated the role of short- and long-term mindfulness-based meditation practice on the subcomponents of the attentional system (alerting, orienting, engagement, and disengagement), and at the early perceptual and non-conscious stages of processing external stimuli that could reflect changes in attentional bias (Brefczynski-Lewis et al., 2007; Chan and Woollacott, 2007; Slagter et al., 2007; Tang et al., 2007; Cahn and Polich, 2009; Hodgins and Adair, 2010; van den Hurk et al., 2010b; Ganaden and Smith, 2011; Vago and Nakamura, 2011). One study that utilized the Attention Network Test (ANT) found significantly better executive monitoring at baseline in a group of experienced meditators (4-week intensive retreat) than control subjects (Jha et al., 2007). After MBSR, individuals demonstrated improved endogenous orienting ability, while the 4-week retreat group showed improved exogenous alerting-related processes, reflecting the advanced training in receptive forms of practice (Jha et al., 2007; Ganaden and Smith, 2011). van den Hurk et al. (2010a,b) used the same ANT task with long-term (~15 years. practice) meditators and found improved orienting and executive monitoring ability in comparison to age-matched controls (van den Hurk et al., 2010a). Five days of IBMT, a receptive practice also demonstrated improvements in executive attention using the ANT task (Tang et al., 2007). Several recent studies have also reported improvements in sustained attention during the performance of other exogenous cueing tasks, or continued performance tasks. One longitudinal study (3 months, 5 h/day) found that retreatants showed enhanced perceptual discrimination and vigilance, an observation that remained constant 3 months after the retreat ended (MacLean et al., 2010). These results suggest a form of plasticity in attention-related networks that can manifest between 5 days and 3 months of intensive mental training.

Alerting attention toward a stimulus can operate at very early levels of perceptual processing (<300 ms) and is sometimes considered an automatic process, taking the form of attentional bias toward, or away from, an object (Mogg et al., 1995). A few hundred milliseconds before an eye movement, visual attention is directed toward the forthcoming target locations, shifting activations in saccade and attention areas of the brain and enabling planning of actions toward those locations (Deubel, 2008). In a sample of female fibromyalgia patients, a preliminary study of mindfulness meditation on attentional bias revealed decreased avoidance at early stages of attention toward pain-related cues in those patients exposed to 8-weeks of mindfulness training (Vago and Nakamura, 2011). Other studies have also showed the influence of meditation practice on early phases of non-conscious attention (Srinivasan and Baijal, 2007; Cahn and Polich, 2009) and bias (Garland et al., 2010; Paul et al., 2012). For example, Srinivasan and Baijal (2007) demonstrated increased mismatch negativity (MMN) amplitudes in concentrative meditation (*Sahaj Samadhi*) practitioners immediately before and after practice, and in contrast to novice practitioners. These studies suggest enhanced perceptual sensitivity even before conscious forms of attention are allocated.

As attentional effort decreases over time and practice (see Figures 3 and 4), the efficiency of attentional networks improves and more cognitive resources are made available. Increased efficiency (decreased effort) of attentional networks has been demonstrated elegantly in reduced resource allocation in one study of subjects who underwent three months of intensive mindfulness meditation training and were tested using an attentional-blink paradigm (Slagter et al., 2007). Attentional blink refers to a deficit in perception of a second target (T2) when presented in rapid succession (<500 ms) following an initial target (T1) embedded in a stream of stimuli presented in close temporal proximity (Slagter et al., 2007). This deficit is believed to result from competition between the two targets for limited attentional resources. A smaller attentional blink and reduced brain-resource allocation to T1 was reflected by a smaller T1-elicited P3b, a brain-potential index of resource allocation (Slagter et al., 2007). Interestingly, those individuals that showed the largest decrease in T1-elicited P3b amplitude generally showed the greatest reduction in attentional-blink size. Another study demonstrated that expert meditators with least hours of experience (10,000–24,000 h) showed increased activity (compared to novices) in the left DLPFC during FA practice (vs. rest), while expert meditators with the most hours of practice (37,000–52,000 h) showed decreased activity in left DLPFC than both groups. Other studies have specifically found improvement in efficiency, reduced interference and increased control. Manna and colleagues (2010) found that right DLPFC deactivation was positively correlated with expertise, suggesting decreased effort in advanced practitioners during FA meditation. Relative meditation experience (ranging from 82 to 19,200 h) has been positively correlated with reduced interference on the Stroop task (Chan and Woollacott, 2007), although Stroop interference was not found in a group of meditators trained in an eight-week MBSR course (Anderson et al., 2007). These data suggest that as meditation practice becomes increasingly effortless in advanced practitioners, more efficient allocation of resources is observed.

### Emotion regulation

There is growing evidence that mindfulness training improves emotion or self-regulation skills as evident from a variety of self-report, physiological, and neuroimaging methods (Baer et al., 2009; Carmody, 2009). One explanatory mechanism underlying alterations in health, brain structure and function may rest on the fact that mindfulness-based meditation practices strengthen neural systems important for emotion regulation, specifically evaluative, expressive, and experiential aspects of emotion. Emotion regulation is therefore also very similar, if not partially redundant with the concept of self-regulation, referring to the ability to shift focus of attention at will, and modulate ongoing emotional activity (e.g., decrease elaborative processing of thoughts/feelings) (Gross, 1998; Northoff, 2005; Carver and Scheier, 2011; Koole et al., 2011). In this context, emotion is measured through multiple components including cognitive, viscerosomatic, behavioral, and physiological responses.

In several publications, Davidson (2000, 2004) has referred to emotion regulation in terms of affective style and has described opportunity for regulation at various dynamics of affective chronometry: (1) the threshold to respond; (2) the magnitude of the response; (3) the rise time to the peak of the response; (4) the recovery function of the response; and (5) the duration of the response. For example, one can consider the recovery function of the response to be rapid (steep) and time to the next response as a refractory phase that may be reduced in practitioners of mindfulness-based meditative techniques. Regulation has been described in the literature through automatic and controlled strategies (Parkinson and Totterdell, 1999; McRae et al., 2011). Automatic regulation refers to the modulation of affect-related variables at a non-conscious level. For example, homeostatic mediators associated with the stress response and involved in generating affect can be non-consciously regulated through training. Controlled regulation can include antecedent-focused or response-focused strategies that require some behavioral or cognitive process. Antecedent-focused strategies focus on controlling the selection or modification of the context to avoid the emotion altogether or to modify the emotional impact (e.g., by performing a secondary distracting task, or cognitive reappraisal) (McRae et al., 2011). Response-focused strategies only affect the output of appraisal process and either augment or suppress behavioral manifestations of one's emotional state, such as smiling, frowning, or experiential avoidance (Gross, 1998; Koole et al., 2011). Many of these controlled strategies may be used in novices when emotional reactivity arises as a form of distraction; however, cognitive strategies are less likely in more advanced practitioners. Non-cognitive strategies are typically encouraged in dealing with the arising and passing of emotions during practice, even when the emotions are particularly intense or stressful. For example, mental noting and labeling the modalities of the emotional experience becomes the first line of defense, a technique shown to reduce amygdala activity and emotional expression (Lieberman et al., 2007). This is more likely an expression of equanimity that naturally arises through continued development of meta-awareness.

The S-ART framework supports the notion that mindfulness-based practice can strengthen controlled emotion regulation processes early on in novice practitioners. Most research in the area of emotion regulation includes a focus on various subdivisions of the PFC that have most consistently been implicated in emotion and cognitive control processes, including decision-making, appraisal, and impulse control. These areas include the OFC, DMPFC, ACC, DLPFC, and VLPFC (Davidson and Irwin, 1999; Ochsner and Gross, 2005; Phillips et al., 2008). Across a number of different MBI studies (Lazar et al., 2005; Holzel et al., 2008; Luders et al., 2009; Grant et al., 2010a; Holzel et al., 2010), differences in GM volume and density (in comparison to non-meditating controls) have been found using MRI in OFC and lateral PFC. Increased activity in prefrontal areas has also been found in experienced meditators (compared to non-meditators) during meditative states (Jevning et al., 1996; Lazar et al., 2000; Holzel et al., 2007, 2011b; Newberg et al., 2010), in some cases specifically in lateral PFC (Baron Short et al., 2010; Farb et al., 2007; Raffone and Srinivasan, 2010). There is some evidence to suggest that abnormal GM concentration and/or functional activity in the left sgACC may be indicative of dysfunctional emotion regulation ability and potential for developing depressive symptomology (Drevets et al., 1997). Other areas found to be critical in their respective interactions with the PFC are the amygdala, hippocampus, striatum (including the nAcc), thalamus, and insula (Davidson and Irwin, 1999; Phillips et al., 2008). The uncinate fasciulus (UF) is a tract of fibers connecting limbic structures to the PFC which is highly plastic over the course of development and critical for emotion regulation and transformation. There is growing evidence that dispositional mindfulness may act as a marker for improved emotion regulation skills (Creswell et al., 2007). For example, increased dispositional mindfulness (measured by the MAAS) has been correlated with increased activation of VLPFC and attenuated activation in the amygdala (Lieberman et al., 2007). Garland and colleagues (2009, 2011) propose that positive reappraisal, a cognitive coping strategy, is a mechanism by which mindfulness functions to regulate emotion and stress. One study supports these claims by demonstrating dispositional mindfulness (assessed by the KIMS) positively correlated with bilateral activation of DMPFC during reappraisal of negative pictures (Modinos et al., 2010). It remains unclear however, to what extent reappraisal is used in mindfulness practice given the emphasis on non-cognitive processing. Other studies have demonstrated evidence for non-appraisal strategies associated with mindfulness (Grant et al., 2010b; Gard et al., 2012). In support of more non-cognitive forms of emotion regulation, reduced activity in executive, evaluative and emotion areas during acute pain (PFC, amygdala, hippocampus) have been found in adept practitioners of Zen, compared to controls (Grant et al., 2010b). Meditators with the most experience showed the largest activation reductions, suggesting a decreased need for effortful control in advanced meditators. Importantly, the lower pain sensitivity and higher thresholds for pain in meditators was strongly predicted by reductions in functional connectivity between executive and pain-related cortices. Results suggest a functional decoupling of the cognitive-evaluative and sensory-discriminative dimensions of pain, possibly allowing practitioners to view painful stimuli more neutrally and with equanimity (Grant et al., 2010b). Grant et al. (2010a,b) also demonstrated increased cortical thickness in dACC that positively correlated with lower pain sensitivity in the Zen meditators, suggesting an ability to monitor and express emotions related to pain without a high negative valence (Grant et al., 2010a).

The S-ART framework also supports the idea that mindfulness-based practice can improve automatic forms of regulation like homeostasis in the face of emotional or physical stress by protecting the internal milieu from the harmful effects of a stressor, which can be referred to as the “raincoat effect,” and by facilitating recovery, which we refer to as the “towel effect.” The raincoat acts as a metaphor for protection, as it protects one from getting wet; the towel acts as a metaphor for recovery, as it facilitates drying off when one already has gotten wet. These metaphors have been used similarly to previous theoretical models for psychoneuroimmunology (Ader et al., 1987; Feder et al., 2009), and are used here to describe the expression of equanimity that is tightly-coupled with the skills of mindful awareness. Equanimity is supported by psychological processes like decentering and non-attachment, de-coupling the sensory and affective components of the stressor. As a result, sympathetic tone is reduced and hypothalamic-pituitary-adrenal (HPA) axis-mediated mobilization is suppressed. A decreased stress response to innocuous cues and rapid return to physiological and emotional baseline in response to real threat should be apparent across all physiological mediators such as the catecholamines (e.g., epinephrine and norepinephrine) from the adrenal medulla, glucocorticoids (e.g., cortisol) from the adrenal cortex, pituitary hormones (e.g., ACTH, prolactin, and growth hormones) and cytokines (e.g., IL-1, IL-6, and TNF-α) from cells of the immune system. Mindfulness training is proposed to reduce the chance for pathophysiological processes or unfavorable psychopathological outcomes by preventing perseverative and chronic responses in the presence or imagined presence of a stress-related physiological challenge and through effective habituation to repeated stress-related challenges (McEwen, 2008). This form of self-regulation prevents “allostatic load,” the cumulative wear and tear on the body and brain due to the overactive or inefficiently managed stress response (McEwen, 1998; Sterling and Eyer, 1988). Long-term adverse effects are avoided, including immunosuppresion, cardiovascular dysfunction and disease, accumulation of abdominal fat, loss of bone minerals, reproductive impairments, decreased neurogenesis, increased neuronal cell death and associated atrophy in the limbic system (Jameison and Dinan, 2001; Sapolsky, 2003; McEwen, 2008).

The connections of the insular cortex with the thalamus, HPA axis and brainstem are thought to provide a mechanism for viscerosomatic and homeostatic feedback to the executive monitor and regulation of sympathetic tone (Critchley, 2005; Craig, 2009). In order to maintain homeostasis, the internal state of the body is critical for the parasympathetic division of the autonomic nervous system (ANS) to respond effectively to stressors. There are two parasympathetic nerve centers in the brainstem medulla: dorsal and ventral. The vagal nuclei promote parasympathetic tone, increased energy conservation through control of cardiovascular and visceral function. The ventral vagal nuclei dampen the sympathetic response specifically by targeting cardiorespiratory organs; whereas, the dorsal nuclei target the gut and associated viscera (Porges, 1995). The S-ART framework predicts mindfulness can facilitate both dorsal and ventral vagal tone through FA on the body in the context of stressors.

There have been a number of studies reporting on the physiological profile of meditators, which appears to be significantly influenced by meditation practice, and may strongly contribute to the neuroimmunological profile as well. For example, studies comparing experienced meditators to controls or short-term meditators have demonstrated a particular physiological profile across styles of practice suggestive of an alert, but hypometabolic state in which there is decreased sympathetic nervous system activity, and increased parasympathetic activity (Young and Taylor, 1998; Benson, 2000; Cahn and Polich, 2006). Across meditation styles, electromyography has revealed muscle relaxation in spite of the upright, unsupported posture (Austin, 2006). Skin conductivity, along with oxygen consumption, heart rate, blood pressure, cortisol, muscle tone, urinary vanillyl mandelic acid (VMA) (a catecholamine metabolite), and rate of breathing all decrease during meditation (Jevning et al., 1992; Benson, 2000; Lazar et al., 2005; Austin, 2006). Critically, this hypometabolic state has been shown to be qualitatively and quantitatively different from simple rest or sleep and more suggestive of a role in prevention of stress-related hypertension or cardiovascular disease (Young and Taylor, 1998; Cahn and Polich, 2006). Other more advanced meditation techniques focus specifically on altering control of breath (e.g., pranayama), and body-temperature (e.g., Tummo), techniques which may be more highly specified toward impacting automatic regulatory processes. During seated meditation, at a middle level of samatha, the breathing rate may drop to only two or three breaths a minute (Lazar et al., 2000; Austin, 2006), while normal adults at sea level breathe about fifteen times per minute. The specific and non-specific effects of mindfulness-based meditation on homeostatic regulation are clearly an aspect of self-regulation that needs further investigation.

Physiological studies have also supported the effects of meditation on non-reactivity related to a rapid change back to baseline after an emotional response. This rapid regulatory mechanism is proposed to be an objective measure of equanimity. For example, experienced transcendental meditators (>2 years experience) showed more rapid decreases in skin conductance following aversive stimuli (Goleman and Schwartz, 1976), while other studies have shown decreased startle amplitude (Delgado et al., 2010; Levenson et al., 2012), and other low-level bottom-up forms of emotion regulation (van den Hurk et al., 2010b). Goldin and Gross (2010) found that social anxiety patients show a more rapid decrease in activation of the amygdala in response to negative self-beliefs after a MBI (Goldin and Gross, 2010). Additionally, Britton et al. (2012) demonstrated more rapid decreases in self-reported state anxiety following a psychosocial stressor (in comparison to pre-MBCT) (Britton et al., 2012). Other studies investigating the effects of meditation on homeostatic regulation have found improved function and effective allocation of inflammatory responses in novice normal meditators and in subjects with major disease diagnoses (e.g., cancer) (Kabat-Zinn et al., 1998; Davidson et al., 2003b; Smith, 2004; Carlson et al., 2007; Pace et al., 2010). For example, Davidson and colleagues (2003b) found that MBSR produced significant increases in antibody titers to influenza vaccine compared with those subjects in a wait-list control group. Interestingly, the magnitude of increase in left-sided anterior PFC activation in EEG predicted the magnitude of antibody titer rise to the vaccine (Davidson et al., 2003b). Pace and colleagues (2010) reported that after 6-weeks of compassion meditation practice, there were reduced innate cytokine (IL-6) and subjective distress responses to a standardized laboratory psychosocial stressor [Trier Social Stress Test (TSST)]. Moreover, individuals with meditation practice times above the median exhibited lower TSST-induced IL-6 and POMS distress scores.

### Extinction and reconsolidation

The elimination of suffering, the end state of mindfulness practice is described often as “stillness of the mind” (Sanksrit: *nirvana*) (Buddhaghosa, 1991). The word “Nirvana” has literally been translated as “blowing out” or “extinction.” In this context, it refers to the extinction of the afflictions (Sanskrit: *klesha*), which prevent happiness and flourishing. (Buddhaghosa, 1991; Analayo, 2003; Bodhi, 1999). Maladaptive habits, distorted perceptions, and biases accumulate through the conditioning or reification of the NS, most of which are not accessible to conscious awareness. The narrative one creates about oneself in terms of self-reflection or future projection becomes increasingly more rigid as it is conditioned over time through a causal chain of repetition (see Figure 1). Each trajectory of self-development represents a repeatedly reconstructed, reinforced, and reified NS with reliable patterns of subject-object relations that are relative stable and accessed during self-specifying processes.

The S-ART framework suggests mindfulness acts as the master self-regulatory mechanism for de-coupling and efficiently integrating experiential and NS modes of processing with the potential to transform the reified self from maladaptive trajectories into more positive, adaptive trajectories. This form of transformation is hypothesized to use circuitry associated with extinction and reconsolidation. Biases of attention and memory related to habitual distortions are proposed to be extinguished and reconsolidated; however, the dosage and quality of meditation time required for such change remains unclear. Although change in constitutionally-based temperament or biological disposition is thought to be less likely than those aspects of self that can be modulated by experience and training (Rothbart and Ahadi, 1994; Kagan, 2003), there is evidence that conditioned fear can be extinguished (Phelps et al., 2004). Exposure, extinction, and reconsolidation are critical mechanisms for reducing habitual anxiety and fear, and in facilitating therapeutic change in past clinical models (Wells and Matthews, 1996; Bishop, 2007; Beck, 2008). Extinction does not erase the original association, but is a process of novel learning that occurs when a memory (explicit or implicit) is retrieved and the constellation of conditioned stimuli that were previously conditioned to elicit a particular behavior or set of behavioral responses is temporarily labile and the associations with each other are weakened through active or passive means. Changing the association contingency of contextual cues and what they mean results in a new memory trace. The new memory trace involves re-consolidated associations of particular contextual stimuli and its previous associations with new stimuli and behavior (Nader et al., 2000; Quirk and Mueller, 2008). The behavioral expression of the new memory is then thought to be in competition with the previously conditioned memory, with particular pathological-oriented biological dispositions proving to increase the time or number of trials necessary for extinction and sensitivity to old fear-based associations (Milad et al., 2007). Extinction and reconsolidation can depend on numerous factors, such as level-of-processing (Craik, 2002), emotional salience (Kensinger and Schacter, 2005), the amount of attention paid to a stimulus (Loftus, 1979), the expectations at encoding regarding how memory will be assessed later (Frost, 1972), or the reconsolidation-mediated strengthening of memory trace (McGaugh, 2000). The closer in time the retrieval occurs to the experience, the more likely weaker components of a memory trace can be reconsolidated given the tendency for weak traces to decay over time (i.e., forgetting). It has been reported that the hypometabolic state of the practitioner may act to facilitate the extinction process by creating novel parasympathetic associations with previously anxiety-provoking stimuli (Cahn and Polich, 2006). The noting and labeling of sensory, cognitive, and emotive states while in a hypometabolic system, such as during OM practice, is also likely to influence the nature of conditioned response toward reconsolidation of more positive trajectories. Awareness alone is proposed to change the conditioned response contingency toward one's patterns of behavior and feelings toward oneself and others, although intentional cognitive processes may also contribute. In association with positive reappraisal strategies, the practitioner may reflect on a particular emotion and decide if it is desirable or undesirable, warranted or unwarranted. If one finds that one would like to change something about the emotional state one is affected by, one can intentionally act to transform the emotions. If, indeed, extinction proves to be a common mechanism for many types of habitual sensory, affective, and motor processing in mindfulness training, one can begin to redefine the solid boundaries and limited interactions between automatic and controlled processing.

In both rodents and humans, the brain areas involved in conditioning and extinguishing fear include the hippocampus, amygdala, rhinal cortices, and VMPFC. Right DLPFC has also been implicated in the extinction of aversive associations, while right VLPFC has been implicated in non-aversive extinction recall (Morgan and Ledoux, 1999). The cerebellum contributes to simple associative learning, while the inferior temporal cortices and dorsal striatum are responsible for conditioning and habitualizing more complex sensory-affective-motor scripts and schemas in visuo-spatial contexts (Buckner and Wheeler, 2001). Structural connectivity between DLPFC and amygdala has also been implicated in learned safety, suggesting effortful control over expression of fear-related behavior (Pollak et al., 2010). The VMPFC appears to modulate the amygdala response and extinguish the expression of fear in this functional circuitry. In fact, cortical thickness of the VMPFC has been shown to positively correlate with extinction recall, and functional connectivity with the hippocampus appears to be related with learned safety (Milad et al., 2007). The DMPFC and dorsal ACC have been shown to be modulatory over the physiological and behavioral expression of fear and thus, may be critical in extinction and reconsolidation as well (Quirk et al., 2010). Dorsal ACC has been shown to be positively correlated with sympathetic activity related to fear expression; whereas, ventral ACC (including pACC and VMPFC) has been more associated with inhibition of expression during extinction, and recall of extinction of fear after extinguishing prior conditioned associations (Etkin et al., 2011).

On a more cellular level, extinction and reconsolidation research has focused on the molecular cascade of events that follows experience and results in reorganization of long-term memory storage. Some lines of investigation have demonstrated that the presence of kinase activity, which persists in order to maintain synaptic potentiation, may be indicative of new memory traces (Wallenstein et al., 2002). Similarly, labeling of epigenetic or transcription factors related to expression of various genes necessary for synaptic plasticity has also been shown to be indicative of extinction and reconsolidation processes (Sweatt, 2009). Several recent studies have shown that extinction learning in rats can be accelerated and strengthened through modulation of these molecular systems as well as through pharmacological noradrenergic and dopamine systems [e.g., D-cycloserine (DCS)] in the mPFC (Quirk et al., 2010).

Although relatively few studies have explored this possibility, there is some evidence that mindfulness-based practices involve exposure, extinction, and reconsolidation processes [see (Holzel et al., 2011a; Treanor, 2011) for review]. Multiple morphometric studies have demonstrated structural changes in the circuitry related to extinction following mindfulness training of as little as 8-weeks (Lazar et al., 2000; Holzel et al., 2008, 2011b; Luders et al., 2009). Cross sectional studies comparing meditators and non-meditators found greater GM concentration in the hippocampus of meditators, suggesting enhanced circuitry for extinction learning and retention (Holzel et al., 2008; Luders et al., 2009). Aside from the changes noted above in memory-related structures, thickness of the mPFC has been found to be directly correlated with extinction retention after fear conditioning, suggesting that its increase in size following training might explain ability in meditators to modulate fear, a mechanism that has yet to be fully explored (Milad et al., 2005; Ott et al., 2010a; Holzel et al., 2011b). Moreover, GM concentration in this region has been correlated with the amount of meditation practice. Holzel and colleagues also recently demonstrated that decreased perceived stress over the 8-weeks of MBSR positively correlated with decreased GM concentration in the right amygdala (Holzel et al., 2011b). Previous research has shown that such morphometric changes are associated with improved emotion regulation and extinction of fear (Milad et al., 2005; Etkin et al., 2011). Functional increases in these regions have also been found during meditation or in the context of emotional probes, suggest a strengthening of the circuitry involved in extinction and a critical role for PFC-hippocampal dynamics in mediating changes in advanced practitioners. We further propose that the posited reductions of negative ruminations about the self reportedly due to mindfulness training (Ramel et al., 2004) may also be mediated by the same functional extinction circuitry.

### Prosociality: improving social cognition

Humans are inherently social beings with a gifted ability for social cognition—to understand others' emotions, intentions, and beliefs. Empathic forms of behavior, including empathy, sympathy, and altruism, have been implicated in conceptual models and theories about social cognition and associated experience sharing and prosocial behavior (Hein and Singer, 2008; Eisenberg et al., 2010; Zaki and Ochsner, 2012). Prosocial behavior is typically defined as voluntary behavior intended to benefit another (Eisenberg et al., 2010). Major facets of prosociality and inter-relationships include forms of mentalizing and perspective taking. For example, imagining the intentions of others has been called “theory of mind” (ToM). Empathy is distinct from ToM and refers to our ability to share the experiences (emotions and sensations) of others. It is believed that mindfulness practice can cultivate a framework for interdependence of self in a social network and include a cognitive framework supporting empathy and mentalizing. In such cases, the conceptual form of meta-awareness (i.e., meta-cognition) allows one to disengage from the contents of awareness and move toward the experiencing of an other's sensory or affective state (Decety and Chaminade, 2003; Singer and Lamm, 2009).

Although prosociality has a biological basis and dispositional differences in empathic responding and prosocial action exist from early life onwards (Rothbart and Ahadi, 1994), patterns of empathic responding and related prosocial behavior are relatively plastic not only during childhood and adolescence, but also during adulthood. Specifically, studies of adults have revealed enhancements in functional activity of a social-cognitive network supporting facets of mentalizing and empathy (Singer and Lamm, 2009; De Greck et al., 2012; Fan et al., 2011). This suggests that some of the neural circuitry underlying empathy can be enhanced through the practice of forms of mental training designed to increase positive affect and prosocial behavior across the lifespan. It is now commonly believed that empathic ability affects the perception and affective style of others' behavior toward oneself (Eisenberg et al., 2010), mutually reinforcing the behavior toward each other. Thus, social and moral cognitions and behavior reflect the nature and valence of self and others' empathic behavior. The S-ART model proposes that plasticity associated with prosociality may be indicative of self-transcendence, dissolving distinctions between self and other and reflecting loving-kindness to both. A greater sense of well-being is likely to depend upon this skill. In fact, altruistic behavior has been associated with greater sense of well-being and acceptance (Lyubomirsky et al., 2005), suggesting all other stated mechanisms can be modulated by the cultivation of prosocial and ethical behavior.

Brain areas that are involved in mentalizing include TPJ, TP, AIC, precuneus, DMPFC, while the ACC, IFG, pSTS, and IPL have been implicated more with prosociality and empathic concern and experience sharing (Singer and Lamm, 2009; Frith and Frith, 2012; Fan et al., 2011; Roy et al., 2012). Imagined self-action relative to imagined experimenter action strongly activates IPL (including TPJ), somatosensory cortex, and precuneus (Ruby and Decety, 2001; Farrer et al., 2003), while Trancranial Magnetic Stimulation (TMS) or lesions of the right TPJ has shown to disrupt the sense of ownership or agency (Tsakiris et al., 2010) and produce dissociative, out-of-body experiences (Blanke and Arzy, 2005). The OMPFC, has been shown to be specifically involved in social modulation of reward value, while the mPFC and TPJ have been shown to be more involved in mentalizing and ToM (Frith and Frith, 2012). The right IPL is more often associated with ToM, whereas the left IPL is more often involved in representing one's own mental states (Decety and Chaminade, 2003). The pSTS (including the TPJ) has been shown to be involved in perceiving biological motion and orienting toward eye gaze (Frith and Frith, 2012). The ACC has been shown to be more involved in a supervisory role and deciphering conflicting information. Posterior IFG has been shown to be involved in emotional judgment and has been suggested to play a role in emotion recognition, predicting emotion, and mirroring action (Frith and Frith, 2012). Fan and colleagues (2011) found that the dorsal ACC and anterior mid-cingulate cortex (aMCC) were recruited more frequently in the cognitive-evaluative form of empathy, while the right AIC was found to be involved in the affective-perceptual form of empathy only, and the left AIC was found to be active in both forms of empathy. Singer and others propose the AIC serves a dual role, a primary mapping of internal states with respect to the self, and the predictive representations or simulation of how the same emotional stimuli feel to others (Singer and Lamm, 2009).

One suggestion to account for overlap between self-other neural substrates has been that understanding the intentions of another is also likely to recruit processes underlying insight into oneself. Interestingly when perceivers passively watch targets experiencing pain or reward, their own engagement of brain areas associated with those states predicts later prosociality (Zaki and Ochsner, 2012). This suggests that increased prosociality may predict decreased differences in the intensity and localization of neural activity in response to one's own experience in contrast to some target individual's experience. This would support a system of experience sharing or prosocial concern; whereas mentalizing may engage separable neural systems. In an experiment by Masten et al. (2010), mentalizing is shown to increase future helping behavior, suggesting a distinct system supporting prosocial behavior through experience sharing and mentalizing. The prosocial system is indicated in Figure 2. Research also indicates that positive social behaviors, specifically maternal nurturing (e.g., licking and grooming), induces positive alterations in the brain and in behavior that promote resilience (Champagne and Curley, 2008). Psychosocial factors, such as decreased levels of denial and avoidant coping behavior, increased levels of social engagement, positive emotion, and dispositional optimism have all been shown to promote resilience (Feder et al., 2009). Collectively these data establish that our brains are continuously shaped both functionally and structurally by experience on which explicit training can capitalize to promote more adaptive brain functioning, especially with relation to prosocial behavior.

In support of the development of prosocial behavior, including mentalizing, empathic concern, and experience sharing, there have been a number of studies to suggest meditators show increased activation of the prosociality circuitry over non-meditators. Lutz and colleagues studied novice and expert meditation practitioners that generated a non-referential loving-kindness-compassion meditation state. To probe affective reactivity, emotional and neutral sounds were presented during the meditation and comparison periods. Concern for others cultivated during this form of meditation enhanced affective processing particularly in response to sounds of distress, and this response to emotional sounds was found to be modulated by the degree of meditation training. The presentation of the emotional sounds was associated with increased pupil diameter and dramatic increased activation of right AIC during meditation (vs. rest) (Lutz et al., 2008a). During meditation, activation in right AIC and mid-insula was greater during presentation of negative sounds than positive or neutral sounds in expert vs. novice meditators. The strength of activation in insula was also associated with self-reported intensity of the meditation for both groups. Insula activity was also strongly coupled to heart-rate, suggesting a relationship between generation of compassion and cardiac function (Lutz et al., 2009). One case study of compassion meditation showed similar activity in areas related to empathic processing (Engstrom and Soderfeldt, 2010). These results and other similar studies that demonstrated neural resonances in shared experience [see (Zaki and Ochsner, 2012)] support the role of limbic circuitry in emotion sharing. The comparison between meditation vs. rest states between experts and novices have shown increased activation in amygdala, right TPJ, and right pSTS in response to all sounds, suggesting greater detection of, and enhanced mentation in response to emotional human vocalizations for experts in comparison to novices during compassion meditation. Interestingly, there was a link between expertise in compassion and the activation in the right pSTS, a finding that is consistent with previous research that indicated pSTS activity predicted self-reported altruism (Tankersley et al., 2007). Increased activity in the amygdala may appear to be a counterintuitive finding to traditional models of controlled emotion regulation, but may be more indicative of regulatory states like equanimity.

Together these data indicate that the mental expertise to cultivate empathic behavior and positive emotion for others' suffering involves a network of prosociality and social cognition.

### Non-attachment and de-centering

One of the advanced outcomes or aspects of insight, developed through mindfulness-based meditation practice is the realization of “no-self,” which stems from the Buddhist teachings describing the nature impermanence (see Section “Defining meditation and mindfulness from the historical perspective”). This form of insight demonstrates that there is no truly existing self (i.e., subject) that continues through life without change and provides the practitioner with the critical distinction between the phenomenological experience of oneself and one's thoughts, emotions, and feelings that appear “thing-like” (Varela et al., 1991). This realization of impermanence of all “thing-like” objects including the self is also described as a “release from mental fixations,” or non-attachment (*Sanskrit: virāga*) (Sahdra et al., 2010). In this sense, mindfulness is a relational process. Mindfulness not only helps to support awareness of the self, it transcends the self-object duality by supporting the realization of the self to be co-dependent with the relations to objects in experience, resulting in a characterization of self as empty and groundless. There may be clear benefits to developing increased awareness of our selves and environment, but the platform from which sustained transformation and insight arise is one that includes the relational quality the practitioner brings toward noting and labeling the modalities of experience—one that is free of the choosing, evaluating, or projecting that is sometimes described as “grasping, aversion, and delusion” (Salzberg, 2011).

Decentering, described as “reperceiving” in past models of mindfulness (Shapiro et al., 2006), is a therapeutic process that introduces a “space between one's perception and response” allowing the individual to disengage or “step outside” one's immediate experience in an observer perspective (i.e., fly on the wall) for insight and analysis of one's habitual patterns of emotion and behavior. Decentering may not necessarily be unique to mindfulness, as it is described in other therapeutic contexts as the “observing self” (Deikman, 1982; Fletcher and Hayes, 2005), or “self-as-witness” (Damasio, 2010). Decentering is compared to clinical constructs such as defusion or psychological distancing (Fletcher and Hayes, 2005; Ayduk and Kross, 2010). It also refers to a process of deautomatization (Ayduk and Kross, 2010), in which there is an undoing of automatic processes that control perception/interpretation. These processes are described to enhance a trait-like ability to shift focus of attention at will, and inhibit elaborative processing of thoughts/feelings. For example, repetitive thinking “I am worthless” vs. recognizing “I am having the thought that I am worthless.” The fusion of self and negative thoughts along with rumination has been shown to play a critical role in exacerbating negative affect, maintaining or heightening anxiety, and increasing cognitive vulnerability to psychopathology (Smith and Alloy, 2009). The insight achieved through mindfulness-based practices provides awareness that one's thoughts are subjective and transient in nature (Safran and Segal, 1990), thus facilitating non-attachment and subsequently improving satisfaction with life, well-being and interpersonal functioning (Sahdra et al., 2010).

Laboratory studies of mindfulness-based meditation practices have yet to identify biological correlates for the psychological constructs of decentering and non-attachment. However, the S-ART framework suggests a significant role for them in relation to practice effects and development of meta-awareness. Some of the extant data suggest functional dynamics between the self-networks may account for the decentering mechanism by which mindfulness training promotes flexibility of information processing between autobiographical and experiential awareness. For example, one study found that the DMPFC is deactivated and negatively coupled to PIC during interoceptive awareness (Farb et al., 2012). Additionally, Farb et al. (2012) found that DMPFC-insula connectivity was absent during exteroceptive focus. Another study investigating adept Zen meditators (in comparison to novices) found stronger connectivity between DMPFC and IPL, an area with both narrative and EPS involvement (Taylor et al., 2012). These data suggest a mechanism by which DMPFC may act to integrate narrative and experiential information, sustain positive activation in interoceptive areas or decouple from them without suppression. Other studies have emphasized areas from the integrative fronto-parietal network as critical for the type of perspective taking required for decentering or non-attachment. For example, one study demonstrated a strong role for dACC in mediating the conscious appraisal of emotional experience (Critchley et al., 2004), while another study showed strong activity in dACC and DMPFC together (Holzel et al., 2007). Future studies will have to clarify the neurobiological mechanisms behind these critical processes.

## Conclusions, challenges, and future directions

Rather than focusing on reducing mindfulness down to a single unitary dimension of cognition, we have attempted to illustrate the complexity of mechanisms by which mindfulness functions to reduce suffering and create a sustainable healthy mind using a framework of self-processing. Suffering is described using both a traditional Buddhist perspective and a contemporary model for psychopathology. Biased self-processing is demonstrated to be a common target for traditional Buddhist and contemporary conceptual models of mindfulness, such that discrepancies in operationalizing mindfulness can find common mechanisms by which mindfulness-based mental training functions. In our attempts to create a unified framework for understanding the mechanisms by which mindfulness functions, we operationalize the concept in two ways: (1) as a broadly defined method for developing a multidimensional skillset that ultimately leads to a reduction in self-processing biases and creates a sustainable healthy mind; (2) A continuous discriminative attentional capacity that we refer to as “mindful awareness”—a skill amongst a set of other skills developed through specific meditation practices. Our interpretation of mindfulness is thus provided by an empirical framework of S-ART to illustrate a method for becoming aware of [and familiar with] the conditions which cause [and remove] distortions or biases in the individual's construction of his or her external or internal experience. Through training in FA, OM, and EE styles of meditation, a sustainable healthy mind is proposed to be supported—reducing maladaptive emotions and cognitions common to most ordinary experience, such as lustful desire, greed, anger, hatred, worry, etc., increasing pro-social dispositions (e.g., compassion, empathy, and forgiveness) toward self and other, reducing attachments to thoughts and feelings, and removing biases inherent in habitual forms of cognition.

The S-ART framework outlines specific neural networks of self-specifying and NS processing along with an integrative fronto-parietal network that is proposed to be supported by six neurocognitive processes developed in mindfulness-based meditation practices. These processes are conceptualized as a skillset proposed to facilitate the integration of self-experience from both top-down and bottom-up mechanisms. It will be critical to examine how these networks change longitudinally through meditation training using advanced multivariate statistical network analyses that also consider changes in attentional and cognitive bias, reduction of psychological symptoms, and first-person self-reports in the neuroimaging acquisition protocols. A neurobiological model (Figure 2) is used to illustrate functional distinctions between the networks; however, gradients of non-conscious and conscious processing must be considered, as functional specification is likely to be graded in nature. The skillset identified herein is an attempt to dismantle the practices that cultivate mindfulness into component mechanisms so that the contemplative sciences can more precisely investigate the dispositional differences amongst practitioners of mindfulness-based meditation practices and practice-specific changes can be objectively correlated with first-person experience.

The challenge in this emerging field continues to be disambiguating the concept of mindfulness from more common usage of the term, and eliminating the “black box” or singular approach to studying meditation. Amongst all the enthusiasm for research and clinical application of mindfulness, there is much foundational basic science and vital clinical trials that remain to be implemented. It is likely that future research in the contemplative sciences will provide a developmental trajectory of different S-ART-specific subcomponents and individual differences across duration and frequency of practice. Although research is beginning to demonstrate the state effects related to a number of contemplative practices, the continued practice of FA and OM meditation induces trait changes that have yet to be adequately and objectively measured longitudinally across progress as a practitioner. It is assumed that one begins receiving benefits of the practices immediately upon initial practice; however, explicit durations of practice and dose effects are issues of concern that should not go unnoticed. As Davidson (2010) emphasizes, it remains uncertain whether study participants can reliably report on the quality and/or magnitude of their practice. These issues are similar for measurement of intensity or quality of practice in long-term practitioners as well. It is also critical that the emerging field examine the adverse effects of long-term practice—effects that typically go unnoticed in clinical trials, but are more prevalent in longer retreats and in practitioners that may or may not have a predisposition toward developing psychopathology. Constructing objective standards for determining extent of expertise and proficiency in a particular meditation technique beyond reported hours of formal practice (i.e., time on the cushion) will have to be developed to support self-reported facets of mindfulness.

It is our hope that this framework will help clarify adaptive mind-brain-body interactions and their therapeutic relevance in the general population and across neuropsychiatric disorders. Although past reviews have focused on the process of mindfulness from a psychological perspective, we have attempted to focus on self-processing using a neurocognitive lens, and distinguish between contemporary and historical interpretations of mindfulness. The S-ART framework also has attempted to disambiguate the term, mindfulness, from other Buddhist concepts (e.g., equanimity, clarity) and integrate them into the multidimensional skillset that is strengthened through FA, OM, and EE practices. Future work that builds off of S-ART and the process models provided will help provide a systematic way to deconstruct the heterogeneity of meditation practices into component parts and illustrate a representative map for the underlying processes of self-transformation. By revealing the neural circuitry and further identifying endophenotypes for mindfulness skill development, the field of contemplative science may help to better predict clinical outcomes and potential targets for the development of biologically based diagnostic and therapeutic strategies for those suffering with mental illness and clarify our understanding related to the nature of mind and consciousness.

### Conflict of interest statement

The authors declare that the research was conducted in the absence of any commercial or financial relationships that could be construed as a potential conflict of interest.
